# Dynamic selection in metallo-organic cube Cd^II^_8_L_4_ conformations induced by perfluorooctanoate encapsulation†

**DOI:** 10.1039/d4sc07105k

**Published:** 2024-11-22

**Authors:** Yu-Qing Li, He Zhao, Ermeng Han, Zhiyuan Jiang, Qixia Bai, Yu-Ming Guan, Zhe Zhang, Tun Wu, Pingshan Wang

**Affiliations:** a Hunan Key Laboratory of Micro & Nano Materials Interface Science, College of Chemistry and Chemical Engineering, Central South University Changsha 410083 China chemwps@csu.edu.cn; b Institute of Environmental Research at Greater Bay Area, Key Laboratory for Water Quality and Conservation of the Pearl River Delta, Ministry of Education, Guangzhou University Guangzhou 510006 China chemwt@gzhu.edu.cn; c Department of Chemistry, The University of Hong Kong Hong Kong SAR 999077 chemjzy@hku.hk

## Abstract

Metallo-organic cages possess flexibility comparable to that of biological receptors and can alter their conformations to better accommodate guest species due to the dynamic reversibility of the coordination bond. Induced fit is widely accepted involving conformation change of the host, while few definitive examples are related to conformation selection. Herein, we report the generation of metallo-organic cube Cd^II^_8_L_4_ with two coexisting conformations, which have been fully confirmed by NMR, ESI-MS and single-crystal X-ray diffraction analysis. The specific guest perfluorooctanoate PFOA selectively binds to the active conformer *C*_2h_-1 to form the PFOA⊂*C*_2h_-1 complex. Furthermore, conformer *D*_2_-2 isomerizes to conformer *C*_2h_-1 in the presence of PFOA, for maximizing the guest binding affinity. This study provides an effective working paradigm for conformation selection, facilitating the understanding of the fundamental mechanism of molecular recognition.

## Abstract 2

Self-assembly of ligand L and Cd^II^ ions affords two metallo-organic cage Cd^II^_8_L_4_ conformers: meta-stable *C*_2h_-1 and thermodynamically favored *D*_2_-2. The inclusion of guest perfluorooctanoate shifts the equilibrium distribution to meta-stable conformer *C*_2h_-1.

## Graphical abstract

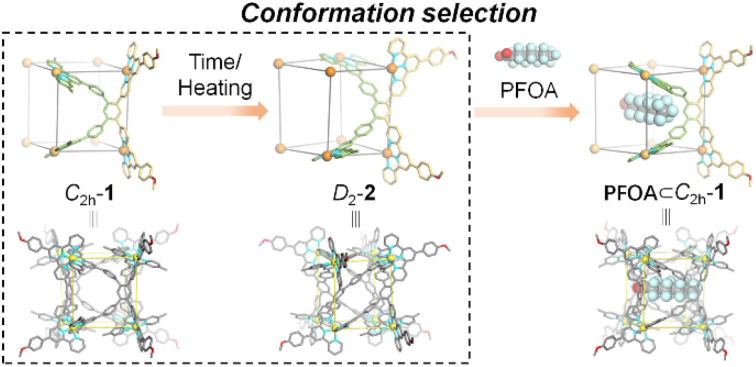

## Introduction

**Fig. 1:**
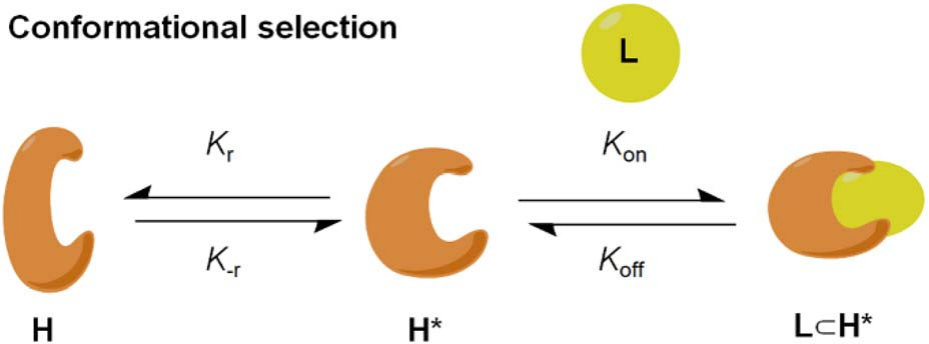
Conformational selection mechanism involving conformational change of the host.

Molecular recognition, described as the binding between a substrate molecule and a protein-host, plays an essential role in various biochemical and physiological functions in an organism.^1,2^ The “conformational selection” hypothesis postulates that there are a series of discrete conformers for receptors in equilibrium (H and H*), in which the substrate molecule selectively interacts with the active one to form a host–guest complex, subsequently shifting the equilibrium distribution of receptor conformers (Fig. 1).^3,4^ It greatly facilitates the comprehensive understanding of molecular recognition in favour of the structure-based drug design, enzymatic catalysis and allosteric regulation of cell signaling.^5–8^ Metallo-organic cages (MOCs),^9–18^ constructed by the coordination between organic ligands and metal ions, serve as an effective model to simulate the molecular recognition of bio-receptors since their characteristic vacant cavities enclose central guest species, enabling various functional applications including chemical separation,^19–21^ sensing,^22–24^ mimic catalysis^25–27^ and luminescent materials.^28–30^

Moreover, the dynamic reversibility of the dative bond within these three-dimensional (3D) metallo-organic structures provides them with recombination ability in response to external stimuli, specifically metallo-organic cages altering their conformation to fit target guests.^31–40^ This guest-induced structural change of metallo-organic cages generally involves an “induced fit” mechanism, in which a guest binds to the receptor in an inactive state, and then undergoes a structural rearrangement into an active model for an optimum fit.^41,42^ However, the molecular recognition of metallo-organic cages based on conformation selection is relatively rare and presents a great challenge. An ideal model for conformational selection should meet the criteria that there are two or more pre-existing conformations in equilibrium and that all of them can be detected.^43,44^ In general, the conformers that coexist possess similar energies, presenting great obstacles for their interconversion. Meanwhile, the complete differentiation of different conformers is still highly challenging due to their structural nuances.^45^

**Scheme 1:**
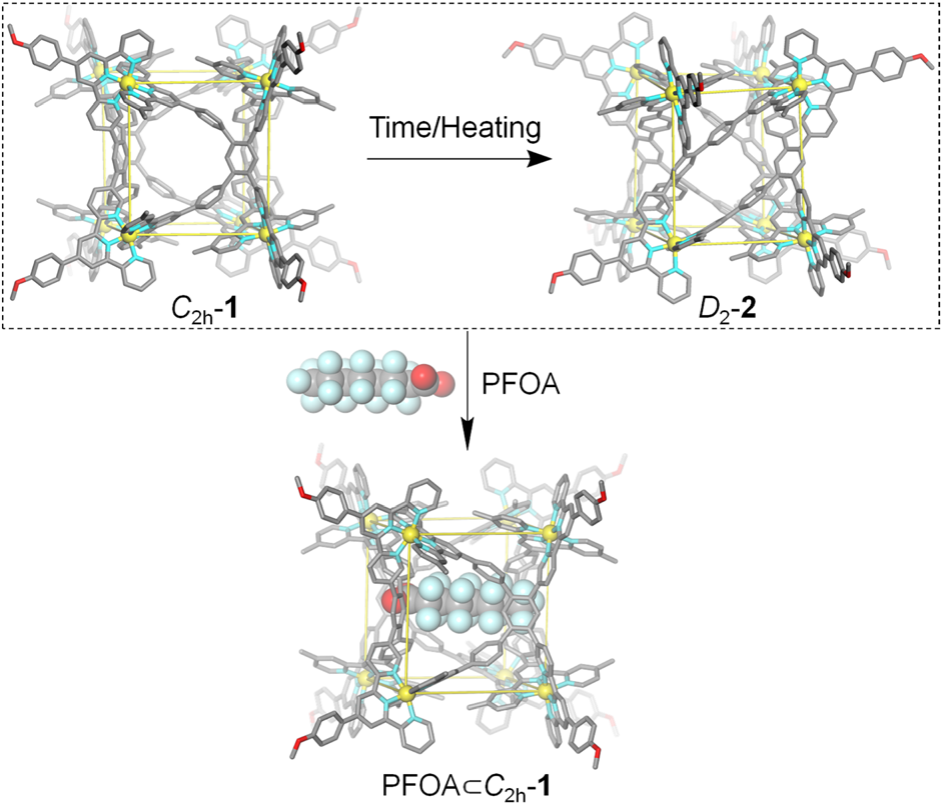
Conformational change of metallo-organic cages Cd^II^_8_L_4_ in response to temperature/time and guest PFOA based on the conformational selection mechanism.

Herein, we report the generation of metallo-organic cages Cd^II^_8_L_4_ in good accordance with the conformation selection mechanism. The self-assembly between tetratopic ligand L and Cd^II^ ions afforded metallo-organic cages Cd^II^_8_L_4_ with the coexistence of two conformers. Conformer 1 is a cuboid metallo-organic cage in which four ligands L cap the equatorial faces with *C*_2h_ symmetry. Differing only in the location orientation of terpyridine units, two parallel ligands L together occupy the equatorial faces of conformer 2, displaying an unprecedented helical cubic structure with *D*_2_ symmetry. Conformer *C*_2h_-1 converts to conformer *D*_2_-2 at a quite slow rate and an elevated temperature can facilitate this process. Owing to the different cavity volume (1: 1263 Å^3^, 2: 867 Å^3^) and shape, the inclusion of guest perfluorooctanoate PFOA, classified as a persistent organic pollutant (POP), can only be achieved by conformer *C*_2h_-1 based on the shape and size complementarity. Furthermore, the addition of PFOA into conformer *D*_2_-2 can shift the equilibrium distribution to conformer *C*_2h_-1 (Scheme 1).

## Results and discussion

**Fig. 2:**
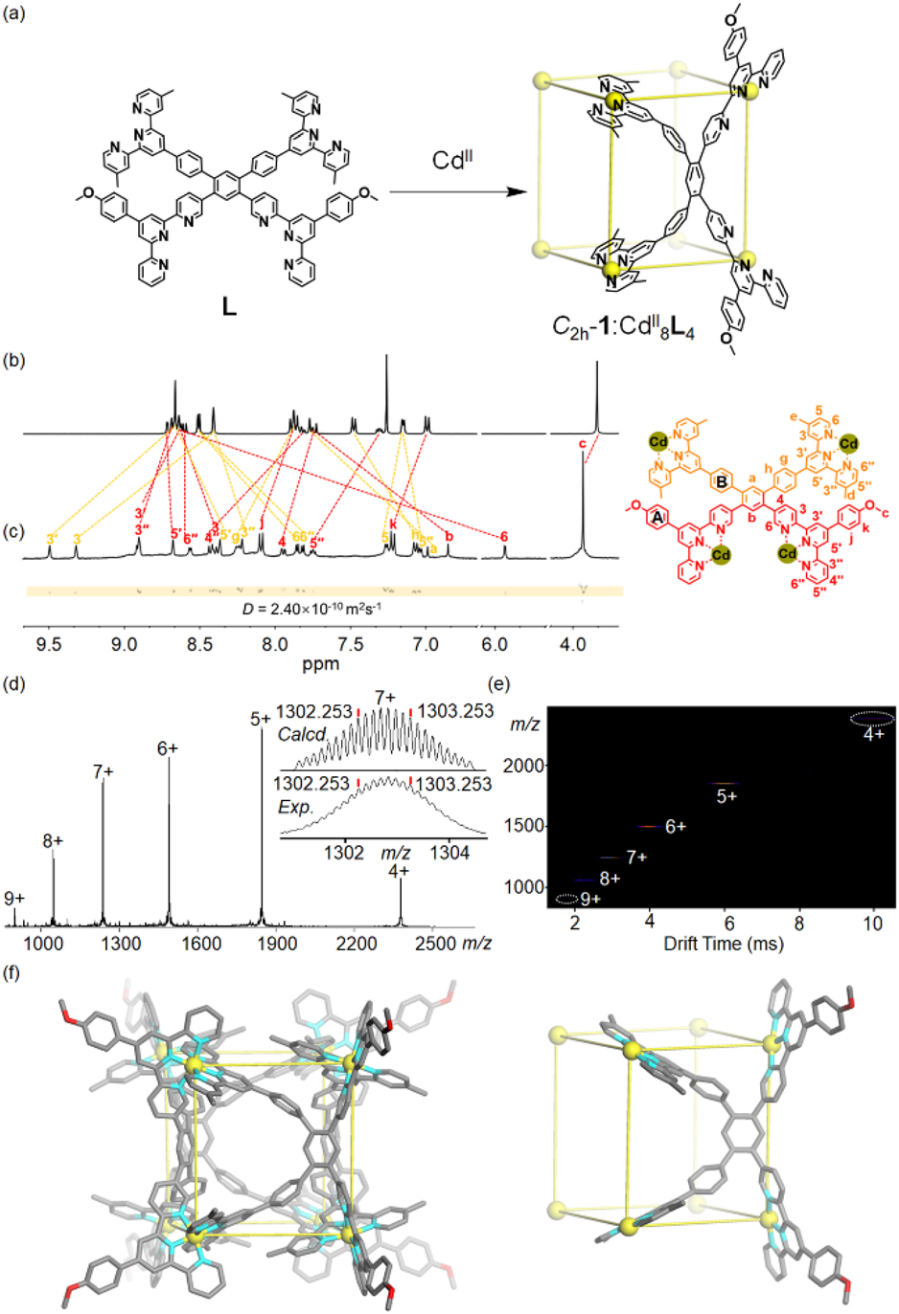
(a) Self-assembly of metallo-organic cage *C*_2h_-1. (b) ^1^H NMR spectrum of ligand L (500 MHz, 298 K, CDCl_3_), (c) ^1^H NMR and DOSY NMR spectra of metallo-organic cage *C*_2h_-1 (500 MHz, 298 K, CD_3_CN-d_3_), (d) ESI-MS with the inset showing the isotopic pattern of charge state 7+, (e) TWIM-MS spectra of metallo-organic cage *C*_2h_-1. (f) Energy-minimized structure and simplified model of *C*_2h_-1.

The tetratopic ligand L contains two kinds of terpyridine units: one connects with the 5-position of the lateral pyridine (part A) and the other is substituted on the *para*-position of benzene attached to the central pyridine (part B) (Fig. 2). It was facilely synthesized through successive Suzuki–Miyaura reactions from commercially available 1,5-dibromo-2,4-diiodobenzene (Scheme S1 and Fig. S1–S13†). Part A and part B possess inwardly converging and outwardly extending conformations, respectively, showing geometric complementarity. So, hetero-connection of parts A and B (tpy^A^-Cd^II^-tpy^B^) is more favourable than homo-connections (tpy^A^-Cd^II^-tpy^A^ and tpy^B^-Cd^II^-tpy^B^) in terms of geometric conformation. Self-assembled supramolecules are normally favoured thermodynamically over oligomeric or polymeric systems because they are synthesized under thermodynamic conditions, facilitating the formation of finite structures at the expense of increased angle strain. In addition, entropy favours closed structures with a minimum number of components rather than polymeric structures, which involve a far larger number of components.^46,47^ So, self-assembly was conducted under thermodynamic conditions: direct reaction of ligand L and two equivalents of cadmium nitrate tetrahydrate Cd(NO_3_)_2_·4H_2_O at 65 °C in a mixed solvent (CHCl_3_ : MeOH = 3 : 4) for 12 h. After cooling to room temperature, excess lithium bis(trifluoromethanesulphonyl)imide (LiNTf_2_) was added to give a precipitate, which was collected by filtration and further washed with D.I. water and MeOH (Fig. 2a and Scheme S2†). The product conformer 1 was quantitatively obtained as a pale white solid after being dried *in vacuo*. The ^1^H NMR spectrum of 1 showed a significant upfield shift of H^6,6′′^ (part B in ligand L) and H^6^ (part A in ligand L), attributed to the characteristic electron shielding effect caused by the pseudo-octahedral bis(terpyridine) complex.^48^ The presence of two singlets of H^3′,5′^ and only one singlet of H^-OMe^ attributed to two kinds of terpyridine moieties strongly demonstrated the equivalent environments of each terpyridine unit, indicating the generation of a highly symmetric species (Fig. 2b, f and S14–S16†). Diffusion-ordered ^1^H NMR spectroscopy (DOSY) analysis confirmed the presence of single discrete species in solution with extracted diffusion coefficient *D* values of 2.4 × 10^−10^ m^2^ s^−1^ (Fig. 2c and S17†). In addition, electrospray ionization mass spectrometry (ESI-MS) was performed to provide composition information of conformer 1. It displays a series of multicharged ions from [Cd^II^_8_L_4_ + 5NTf_2_^−^ + 2NO_3_^−^]^9+^ to [Cd^II^_8_L_4_ + 10NTf_2_^−^ + 2NO_3_^−^]^4+^, demonstrating the exclusive formation of Cd^II^_8_L_4_ type assemblies. It's noted that the experimental isotopic patterns for each charge state agreed well with the calculated distributions (Fig. 2d and S18†). 2D travelling wave ion migration mass spectrometry (TWIM-MS) exhibited a narrow drift time distribution of each charge state for conformer *C*_2h_-1, ruling out other isomers and conformers (Fig. 2e). The results of NMR, MS and computational simulation strongly indicate a metallo-organic cube with *C*_2h_ symmetry of conformer 1. It's noted that self-assembly between ligand L and Zn^II^ gives an isostructural metallo-organic cage [Zn^II^_8_L_4_] (Fig. S43 and S44†).

**Fig. 3:**
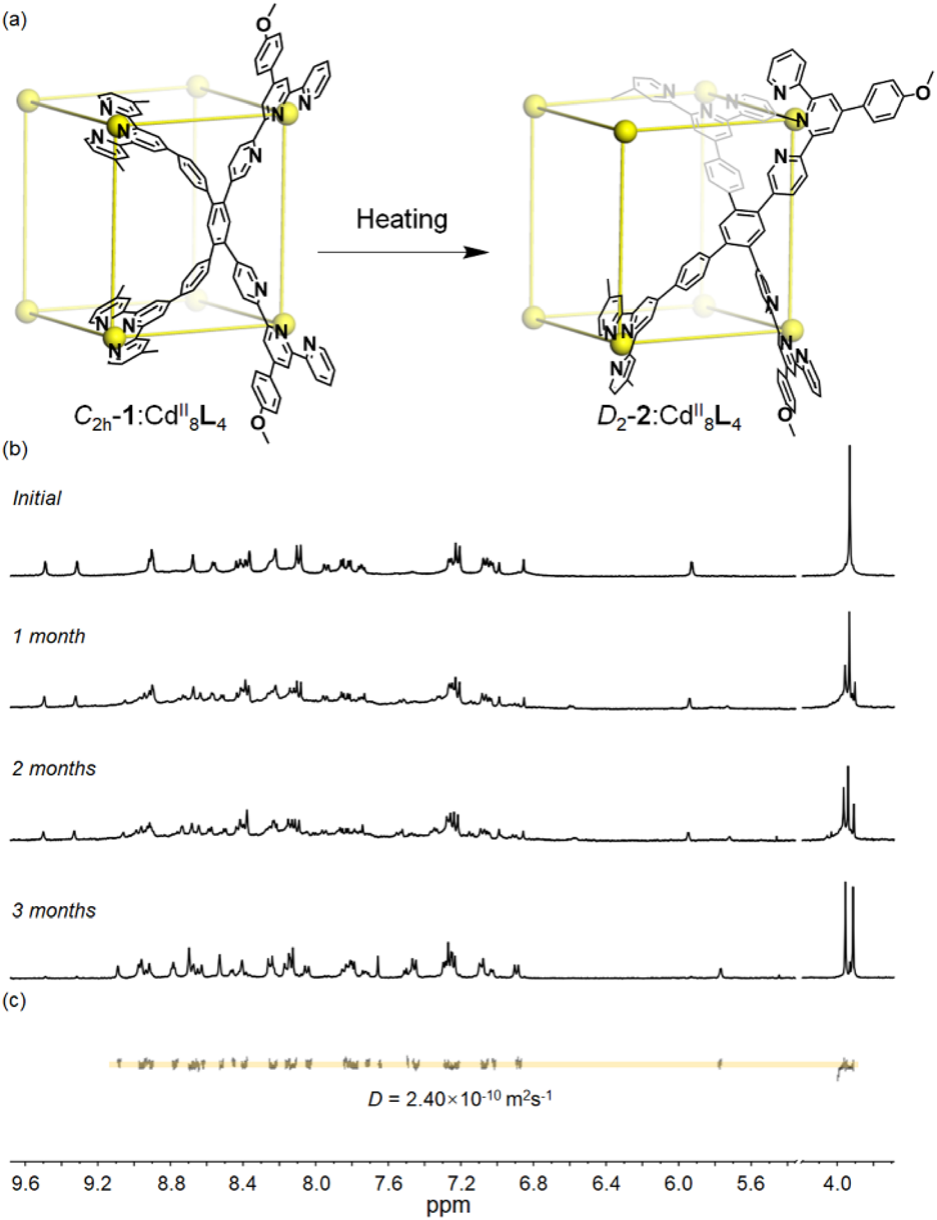
(a) Conformational conversion from conformer *C*_2h_-1 to conformer 2. (b) Time-resolved ^1^H NMR spectra, and (c) DOSY NMR spectra of metallo-organic cage conformer 2 (500 MHz, 298 K, CD_3_CN-d_3_).

As time went by, an unexpected growth in the number of proton resonances was observed in the ^1^H NMR spectra of *C*_2h_-1. Three months later, a brand-new and highly complicated ^1^H NMR spectrum can be detected, showing four sets of terpyridine signals in the aromatic region along with two singlets derived from methoxy (Fig. 3b and S19–S21†). Its ^1^H DOSY NMR spectrum confirms that all signals have the same diffusion coefficient *D* of 2.4 × 10^−10^ m^2^ s^−1^ which is similar to that of conformer *C*_2h_-1 (Fig. 3c and S22†). The identical Cd^II^_8_L_4_ composition of metallo-organic cage 2 was further verified by ESI-MS coupled with TWIM-MS (Fig. S23 and S24†). Therefore, it can be concluded that the product self-assembled from ligand L and Cd^II^ ion has two conformers (Fig. 3a) with different symmetries and thus different peak patterns in the ^1^H NMR spectra. Conformational conversion from conformer *C*_2h_-1 to conformer 2 is slow on the NMR timescale, enabling their direct differentiation in ^1^H NMR spectra. In order to accelerate conformational conversion, the NMR tube of *C*_2h_-1 was heated at 65 °C, achieving a complete transformation to conformer 2 after four weeks. It's noted that conformer *C*_2h_-1 cannot be recovered even by freezing treatment of conformer 2 (208 K, 1 month). The above results illustrate that conformer 2 is thermodynamically preferred compared to metastable and kinetic conformer *C*_2h_-1.

**Fig. 4:**
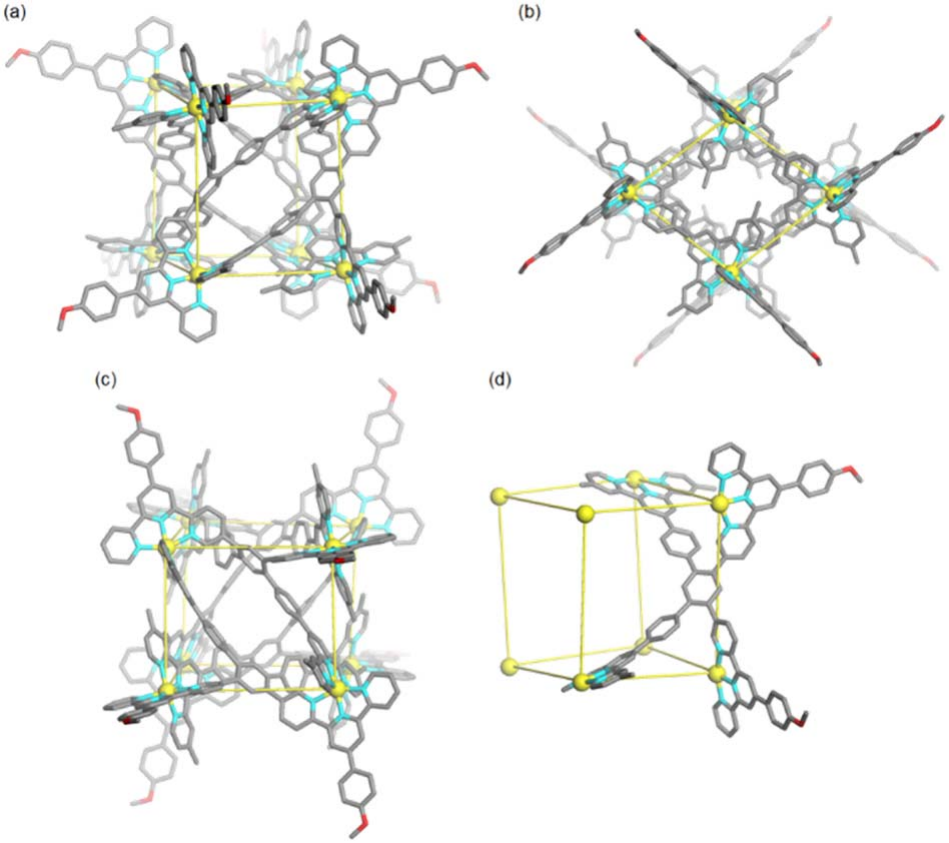
(a–c) Three views of the single crystal X-ray structure of metallo-organic cage *D*_2_-2. (d) The geometrical illustration of metallo-organic cage *D*_2_-2.

Subsequently, by slow diffusion of isopropyl ether over a month or three days' diffusion of toluene into an acetonitrile solution of the product assembled from ligand L and Cd^II^ ions, single crystals suitable for X-ray diffraction (SC-XRD) were achieved (Table S1†). There are two problems in obtaining high-quality crystals: (1) during the crystallization process, gel is always generated and (2) due to the presence of large voids and highly disordered solvents/anions, the reflections in the high *θ* angle are too weak to obtain completeness and good data/parameter ratios. Fortunately, the satisfactory refinement results are sufficient for the cage structure determination. It revealed a helical cuboid structure in which the eight Cd^II^ ions occupy the vertices. The neighbouring Cd^II^⋯Cd^II^ distances were measured to be 11.4–13.5 Å corresponding to the different edges of the cube (Fig. S35†). Along the equatorial plane of the cubic structure, three terpyridine arms (two tpy-A, one tpy-B) in ligand L bridge the metal centers and the last one (tpy-B) extends to the opposite plane and coordinates to the metal center on the body diagonal. In this manner, two ligands L together form a face of the helical cube which is totally different from the ligand face-capped cuboid metallo-organic cages.^49,50^ The solid-state structure of the helical cube shows multiple extended orientations of terpyridine units which is consistent with the increased number of proton resonances in the ^1^H NMR spectra of conformer *D*_2_-2 rather than highly-symmetric conformer *C*_2h_-1 with only two kinds of terpyridine units. There are two *C*_2_ axes that are perpendicular to each other and no symmetry plane can be observed, demonstrating the *D*_2_ symmetry of helical cube 2 (Fig. 4). Despite many attempts, only single crystals attributed to *D*_2_-2 can be detected, probably because it's easier to crystallize in contrast to *C*_2h_-1.

As we fully confirmed the conformational conversion from *C*_2h_-1 to *D*_2_-2 and their exact structures, calculations using Forcite of Materials Studio were performed to investigate the energy difference between these conformers. Conformer *D*_2_-2 is 28.19 kcal mol^−1^ more stable than conformer *C*_2h_-1 (Fig. S39†), rationalizing the thermodynamically controlled conversion. The cavities of the two conformers (*C*_2h_-1 and *D*_2_-2) were well-enclosed by ligand L, and the methyl groups of part B further blocked the pores of the two apical vacant planes (Fig. 4b and S36†). Calculated using VOIDOO,^51^ the internal cavity volumes of two conformers were different, at 1263 Å^3^ and 867 Å^3^ for *C*_2h_-1 and *D*_2_-2, respectively (Fig. S37 and S38†).

**Fig. 5:**
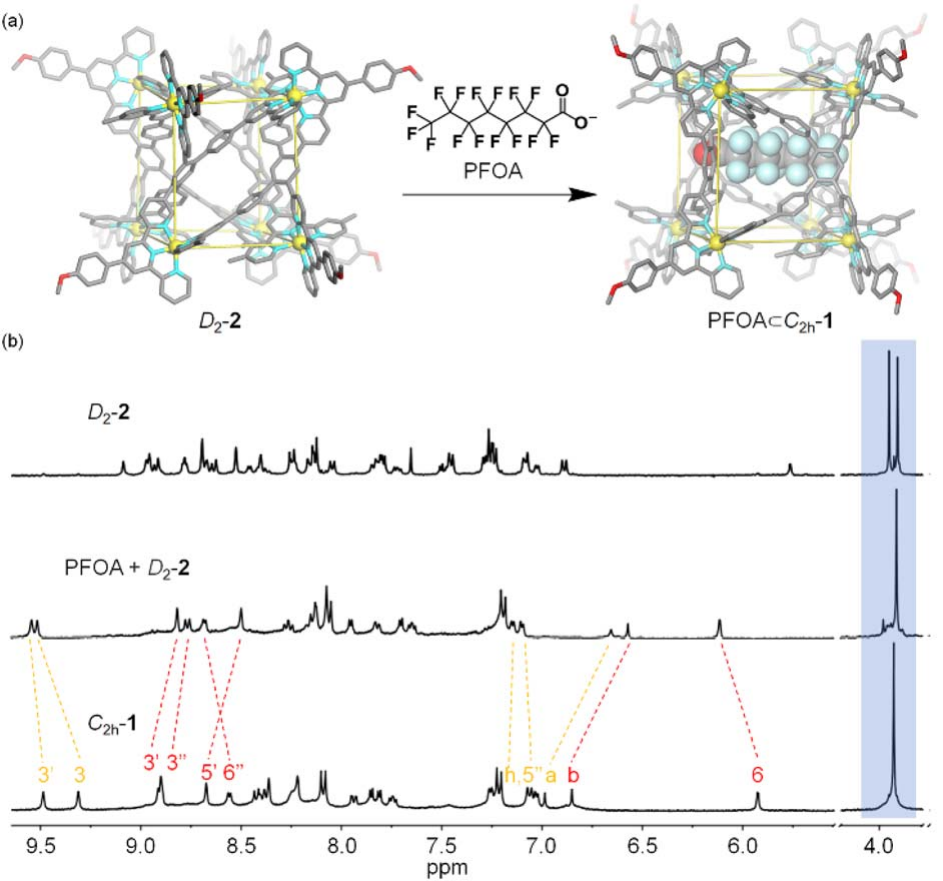
(a) PFOA-induced conformational selection from *D*_2_-2 to PFOA⊂*C*_2h_-1. (b) ^1^H NMR spectra of (top) *D*_2_-2, (middle) *D*_2_-2 + PFOA, (bottom) *C*_2h_-1 (500 MHz, CD_3_CN-d_3_, 298 K).

The two conformers *C*_2h_-1 and *D*_2_-2 may possess drastically different binding affinities toward the same guest due to their different cavity size and shape. So, it's an ideal model for conformational selection, in which a guest will only be encapsulated by one conformation and will not bind to the other at all. Moreover, when accommodating a specific guest, the original equilibrium between the conformers will be broken, leading to the exclusive generation of one kind of host–guest complex owing to the dynamic reversible nature of dative bonds. After screening (Fig. S25–S28†), perfluorooctanoate (PFOA) was found to be a suitable guest. PFOA accumulates in water resources and poses serious environmental and health threats due to its non-biodegradable nature and long environmental persistence time.^52–54^ Effective recognition of PFOA by artificial hosts may bring about the sensing or even adsorption materials for PFOA pollutant.^55–57^ The addition of excess PFOA into the solution of conformer *D*_2_-2 resulted in a reduction in the number of terpyridine units from 4 to 2, along with the appearance of only one singlet assigned to methoxy for part A of ligand L, strongly indicating the main structure of conformer *D*_2_-2's conversion to conformer *C*_2h_-1. And, compared to the ^1^H NMR spectra of conformer *C*_2h_-1, proton resonance of H^3^ on part B of ligand L displayed a moderate downfield shift (from 9.31 ppm to 9.51 ppm) and the other signals shifted to the upfield. In addition, ^19^F NMR signals of the fluorine atoms on *C*_2h_-1 derived from NTf_2_^−^ showed obvious upfield shift with the addition of PFOA, along with the upfield shift and broadening of ^19^F signals on guest PFOA, which is consistent with previous reports.^58,59^ The obvious changes in ^1^H and ^19^F NMR spectra were indicative of a molecule of PFOA encapsulated within host *C*_2h_-1 (Fig. 5b and S29–S31†). In addition, ESI-MS results exhibited a series of multicharged ions from [Cd^II^_8_L_4_ + PFOA + 7NTf_2_^−^ + KNO_3_]^8+^ to [Cd^II^_8_L_4_ + PFOA + 9NTf_2_^−^ + KNO_3_]^6+^, indicating the presence of host–guest complex PFOA⊂*C*_2h_-1 (Fig. S32†). That is, as the cavity of conformer *D*_2_-2 could not provide a suitable environment for PFOA, it transformed into conformer *C*_2h_-1 with the aim to accommodate the guest. Thus, the binding mechanism can be unambiguously assigned to conformational selection (Fig. 5a).

To further understand the mechanism of guest-induced conformational selection, theoretical calculations were conducted by using Forcite of Materials Studio (molecular-level interactions of the host–guest complex are shown in the ESI†). The calculated results are in good agreement with the experimental phenomenon, that is, the binding energy of host–guest complex PFOA⊂*C*_2h_-1 is −31.5 kcal mol^−1^, which is more than that of PFOA⊂*D*_2_-2 (Δ*E* = −7.9 kcal mol^−1^) (Fig. S40–S42†). It's noted that the addition of PFOA into the solution of *C*_2h_-1 afforded the same result as PFOA⊂*C*_2h_-1. In the ^1^H and ^19^F NMR titration experiments performed by continuously adding PFOA into *C*_2h_-1, no free host *C*_2h_-1 was detected, suggesting PFOA's fast exchange binding model on the NMR timescale (Fig. S29†). The 1 : 1 host–guest stoichiometry was confirmed by the Job plot method (Fig. S33†).^60^ The host–guest binding constant (*K*_a_) was estimated by ^1^H NMR titration and calculated to be 690 ± 20 M^−1^ based on Bindfit (Fig. S34†).^61^

## Conclusions

We have presented a metallo-organic cube Cd^II^_8_L_4_ with two discrete conformations based on the different location orientations of tetratopic ligand L. NMR, ESI-MS and SC-XRD techniques clearly supported the coexistence of two conformers. Time-resolved ^1^H NMR spectra confirmed conformer *C*_2h_-1's extremely slow conversion to conformer *D*_2_-2. Due to the different cavity volume and shape of the two conformers, a specific guest PFOA was selectively encapsulated by *C*_2h_-1. Moreover, in the presence of PFOA, the conformational equilibrium between *C*_2h_-1 and *D*_2_-2 can be shifted to conformer *C*_2h_-1 with the aim to maximize the binding affinity. This host–guest behaviour strictly follows the conformation selection model, which can serve as a standard paradigm to study the deep mechanism of molecular recognition. In addition, the metallo-organic cube provides a suitable host, possessing the potential as a sorbent material and phase transfer or extraction system for the PFOA pollutant. Further studies will concentrate on improving the binding affinity through reasonable modifications of metallo-organic cubic cages.

## Data availability

Crystallographic data for *D*_2_-2 have been deposited at the CCDC database under CCDC number 2359196† and can be obtained from The Cambridge Crystallographic Data Centre *via*https://www.ccdc.cam.ac.uk/data_request/cif. Further analytical data are reported in the ESI† to this article. Data are available upon request from the authors.

## Author contributions

T. Wu and J. Z. conceived the study. Y.-Q. Li and Z. H. performed the synthesis. E. Han and Q. Bai performed characterization of the materials. Y.-M. Guan and Z. Zhang assisted in structural characterization (X-ray, NMR, and MS analyses). T. Wu wrote the original draft. P. Wang reviewed and edited the paper.

## Conflicts of interest

There are no conflicts to declare.

## Supplementary Material

SC-OLF-D4SC07105K-s001

SC-OLF-D4SC07105K-s002
