# Supplementary material for: Dynamic selection in metallo-organic cube CdII8L4 conformations induced by perfluorooctanoate encapsulation
Source: Chem Sci. 2024 Nov 22;16(1):364–70. doi: 10.1039/d4sc07105k (PMC11604167; doi:10.1039/d4sc07105k)
Supplement: SC-016-D4SC07105K-s001 [file SC-016-D4SC07105K-s001.pdf]

Supplementary Information (SI) for Chemical Science.
This journal is © The Royal Society of Chemistry 2024

# Supporting Information

## Dynamic selection in metallo-organic cube $Cd^{II}_8L_4$ conformations induced by perfluorooctanoate encapsulation

Yu-Qing Li,[a,#] He Zhao,[a,#] Er-Meng Han,[a] Zhiyuan Jiang,[c,*] Qixia Bai,[b] Yu-Ming Guan,[b] Zhe Zhang,[b] Tun Wu,[b,*] Pingshan Wang[a,*]

# Table of Contents


1. **General Procedures** ........................................................................................3

2. **Synthesis of the Ligands L and metallo-organic cages $Cd^{II}_8L_4$.** ....................................5

3. **NMR and MS spectra data of Compound and supramolecule.** ....................................9

4. **Host-guest complexation** ........................................................................................23

5. **Single-crystal X-ray diffraction (XRD)** ........................................................................29

6. **Volume Calculations**........................................................................................31

7. **Calculation** ........................................................................................32

8. **The NMR and MS spectra date of $Zn_8L_4$** ........................................................................35

9. **References**........................................................................................36

## 1. General Procedures

All starting materials were purchased from Aldrich and Alfa Aesar, and used without further purification. Compound **1** and **3** were synthesized according to the reported methods[S1, S2]. Column chromatography was conducted using basic $Al_2O_3$ (Sinopharm Chemical Reagents Co. Ltd, 200-300 mesh) or $SiO_2$ (Qingdao Haiyang Chemical Co., Ltd, 200-300 mesh) and the separated products were confirmed by NMR spectra using a Bruker Avance 400-MHz or 500-MHz NMR spectrometers in $CDCl_3$, and, $CD_3CN$ with a TMS standard. Electro-spray ionization (ESI) mass spectra were recorded with a Bruker microOTOF-QII or a Waters Synapt HDMS G2 instrument, using solutions of 0.01 mg/mL in $CHCl_3$/MeCN (1:3, v/v) for ligands and 0.2 mg/mL in MeCN or MeCN/MeOH (3:1, v/v) for complexes.

**NMR.** Nuclear magnetic resonance spectra data were recorded on a Bruker 500 MHz, a Bruker 500 MHz and a 400 MHz nuclear magnetic resonance instrument using, $CDCl_3$ and $CD_3CN$ as the solvents with tetramethylsilane (TMS) as the internal standard at 25 °C.

**ESI-MS** and **TWIM-MS.** ESI mass spectrometry and traveling wave ion mobility (TWIM) experiments were conducted on a Waters Synapt HDMS G2 instrument with a LockSpray ESI source, using the following parameters: ESI capillary voltage, 1.3-3.0 kV; sample cone voltage, 20-25 V; extraction cone voltage, 1.1-3 V; desolvation gas flow, 800 L/h ($N_2$); trap collision energy (CE), 4 V; transfer CE, 0 V; trap gas flow, 2.0 mL/min (Ar); source temperature, 30 °C; and desolvation temperature, 30 °C. All samples were dissolved in $CH_3CN$ or $CH_3CN/CH_3OH$ (1:1, v/v) and then infused into the -100, KD Scientific). For TWIM experiments, the helium cell gas flow was held at 180.0 mL/min and the ion mobility cell gas flow was held at 90.0 mL/min ($N_2$). The TWIM DC traveling wave velocity and height were set as 683 m/s and 26.3 V, respectively. Data were collected and analyzed by using MassLynx 4.1 and DriftScope 2.4 (Waters).

**UV-vis absorption**. UV-vis absorption spectra were recorded on a Thermo Fisher Scientific Evolution 201 spectrophotometer at room temperature ($10^{-6}$ M in acetonitrile) and were corrected with the background spectrum of the solvent.

**Single crystal X-ray diffraction**. Single-crystal X-ray diffraction data for $D_2$-**2** (CCDC: 2359196) w as collected on a Bruker D8 VENTURE METALJET liquid metal Xray source system (GaKα, λ= 1.3 4138 Å) at low temperatures (100 K), equipped with an Oxford 800 Plus liquid nitrogen vapor coolin g device. Using Olex2,[S4] the structures were solved with the SHELXT [S5] structure solution program u sing Intrinsic Phasing and refined with the SHELXL[S6] refinement package using Least Squares mini misation. The contribution of disordered guest molecules to scattering was removed by the SQUEEZ E[S7] program of PLATON.[S8]

**Energy calculation method**: First-principles calculations are performed by Forcite at Materials Studio. Simulation is performed using a universal force field. The containment limits for energy, maximum force, and maximum displacement are set to $2*10^{-5}$ kcal/mol, 0.001 kcal/mol/Å, $e^{-5}$ Å, respectively. The effects of the counterions were omitted in the modeling. Structures were constructed on the basis of the crystal structures. Valence energy (diag. terms) contains the contributions of bond, angle, torsion, and inversion. Valence energy (cross terms) contains the contributions of stretch-stretch, stretch-bend-stretch, stretch-torsion-stretch, separated-stretch-stretch, torsion-stretch, bend-bend, torsion-bend-bend and bend-torsion-bend. Non-bond energy contains the contributions of van der Waals and electrostatic interactions. All of the energies are reported in kcal/mol.[S9-S10]

## 2. Synthesis of the Ligands L and metallo-organic cages $Cd^{II}_8L_4$.

Compound **1** were synthesized according to the reported methods[S1].

Compound **3** were synthesized according to the reported methods[S2].

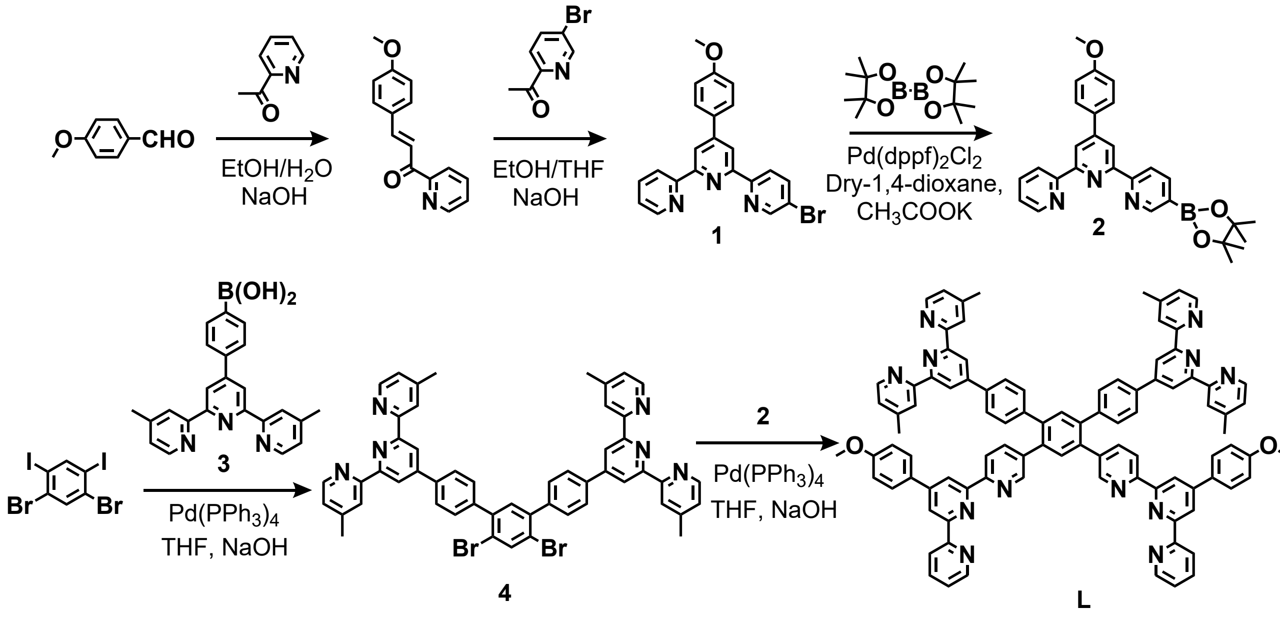


**Scheme S1:** The synthetic route of ligand **L**.

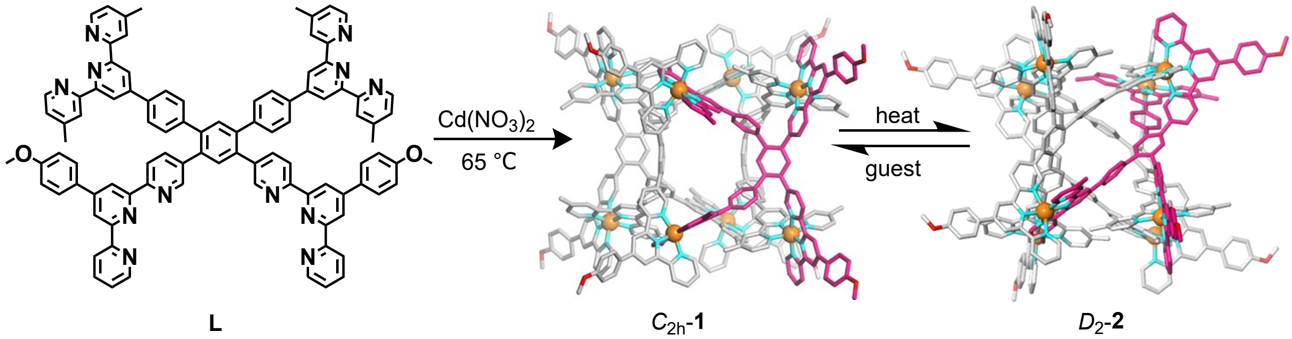


**Scheme S2:** The synthetic route of metallo-organic cages $Cd^{II}_8$**L**$_4$ ($C_{2h}$-**1** and $D_2$-**2**).

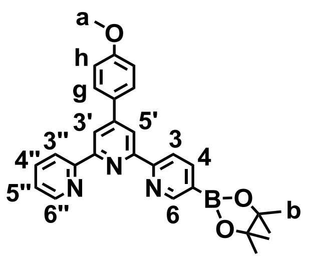


**Compound 2:** Mixing compound **1** (1.3 g, 3.0 mmol) and Bis(pinacolato)diboron (0.9 g, 3.6 mmol) into a 100 mL flask, then 1,4-Dioxane (50 mL), and $CH_3COOK$ (0.9 g, 9.0 mmol) was added. The system was degassed for 10 min, and $Pd(dppf)_2Cl_2$ (150 mg, 0.2 mmol) as the catalyst was added. The mixture was stirred at 85 °C under nitrogen for 8 h, after cooled to ambient temperature. Concentrated

in vacuo followed by adding $CH_2Cl_2$ to dissolve the product, filtered, then removed $CH_2Cl_2$ under vacuum to give the product, as white solid: 1.3 g, 93%. $^{1}$H NMR (400 MHz, $CDCl_3$) δ 8.98 (s, 1H, tpy-$H^{3'}$), 8.69 (s, 1H, tpy-$H^{5'}$), 8.69-8.64 (m, 2H, tpy-$H^{6,6''}$), 8.62-8.60 (d, 1H, *J*= 8 Hz, tpy-$H^{3}$), 8.58-8.56 (d, 1H, *J*= 8 Hz, tpy-$H^{3''}$), 8.18-8.16 (d, 1H, *J*= 8 Hz, tpy-$H^{4}$), 7.84-7.81 (m, 3H, tpy-$H^{4''}$, Ph-$H^{g}$), 7.30-7.27 (m, 1H, tpy-$H^{5''}$), 6.98-6.96 (d, 2H, *J*= 8 Hz, Ph-$H^{h}$), 3.82 (s, 3H, $H^{a}$), 1.33 (s, 12H, $H^{b}$). $^{13}$C NMR (126 MHz, $CDCl_3$) δ 160.54, 158.29, 156.37, 155.90, 155.83, 155.05, 149.73, 149.08, 143.13, 136.86, 130.76, 128.55, 123.75, 121.42, 120.44, 118.77, 118.53, 114.35, 84.23, 55.37, 24.91. ESI-MS (465.4 calcd. For $C_{28}H_{28}BN_3O_3$): *m/z* 466.231 $[M + H^+]^+$ (calcd *m/z*: 466.231).

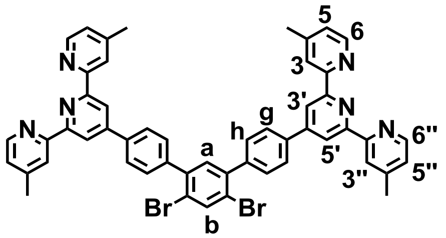


**Compound 4:** 1,5-dibromo-2,4-diiobenzene (3.0 g, 6.2 mmol) and compound **3** (4.5 g, 12.4 mmol) was added to a 500 mL flask, then THF (200 mL) and NaOH (1.5 g, 37.2 mmol) in 30 mL of water was added. The system was degassed for 10 min, and $Pd(PPh_3)_4$ (346.0 mg, 0.3 mmol) as the catalyst was added. The mixture was stirred at 65 °C under nitrogen for 2 d, after cooled to ambient temperature, then concentrated in vacuo followed by column chromatography ($Al_2O_3$), eluting with the mixture of petroleum ethe and $CH_2Cl_2$ (v/v, 1:3) to pure the product, as white solid: 3.0 g, 54%. $^{1}$H NMR (400 MHz, $CDCl_3$) δ 8.76 (s, 4H, tpy-$H^{3',5'}$), 8.59-8.58 (d, 4H, *J*= 4 Hz, tpy-$H^{6,6''}$), 8.47 (s, 4H, tpy-$H^{3,3''}$), 8.05 (s, 1H, $H^{b}$), 8.00-7.98 (d, 4H, *J*= 8 Hz, Ph-$H^{g}$), 7.61-7.59 (d, 4H, *J*= 8 Hz, Ph-$H^{h}$), 7.43 (s, 1H, $H^{a}$), 7.19-7.18 (d, 4H, *J*= 4 Hz, tpy-$H^{5,5''}$). $^{13}$C NMR (101 MHz, $CDCl_3$) δ 156.21, 155.96, 149.54, 149.00, 148.09, 141.33, 140.32, 138.12, 136.89, 133.06, 129.86, 127.15, 124.89, 122.15, 121.96, 119.04, 21.40. ESI-MS (906.7 calcd. For $C_{52}H_{38}Br_2N_6$): *m/z* 907.161 $[M + H^+]^+$ (calcd *m/z*: 907.151).

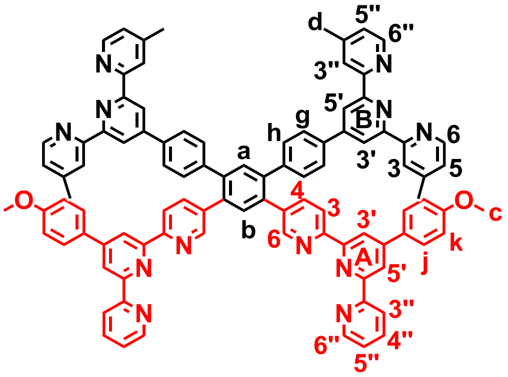


**Compound L**: To a solution of **4** (1.5 g, 1.7 mmol) and **2** (2.1 g, 4.4 mmol) in 140 mL THF, NaOH

(408 mg, 10.2 mmol) was added. The mixture was degassed for three times and backfilled with Argon, then $Pd(PPh_3)_4$ (230.0 mg, 0.2 mmol) was added. After refluxing for 2 d, the mixture was cooled to 25 °C and poured into $CH_2Cl_2$ solvent. Collecting organic layers, and then the aqueous layer was extracted by $CH_2Cl_2$. The combined organic phase was washed with brine and dried with $Na_2SO_4$. After concentration in vacuo, the residue was purified by flash column chromatography ($Al_2O_3$ 200-300 mesh), eluting with $CH_2Cl_2$: $CH_3OH$ (v/v, 100/0.25) to give **L**, as a white solid: 1.7 g (73%). $^1$H NMR (600 MHz, $CDCl_3$) δ 8.75-8.74 (d, 2H, *J*= 6 Hz, A-tpy-$H^{3'}$), 8.73-8.72 (m, 6H, A-tpy-$H^{5'}$, B-tpy-$H^{3',5'}$), 8.70-8.69 (m, 4H, A-tpy-$H^{3}$, A-tpy-$H^{6}$), 8.68-8.67 (d, 2H, *J*= 6 Hz, A-tpy-$H^{3''}$), 8.64-8.63 (d, 2H, *J*= 6 Hz, A-tpy-$H^{6''}$), 8.55-8.54 (d, 4H, *J*= 6 Hz, B-tpy-$H^{6,6''}$), 8.45 (s, 4H, B-tpy-$H^{3,3''}$), 7.90-7.89 (d, 4H, *J*= 6 Hz, A-Ph-$H^{j}$), 7.88-7.87 (d, 4H, *J*= 6 Hz, B-Ph-$H^{g}$), 7.84-7.81 (t, 2H, *J*= 9 Hz, A-tpy-$H^{4''}$), 7.79 (s, 1H, $H^{a}$), 7.77-7.76 (d, 2H, *J*= 6 Hz, A-tpy-$H^{4}$), 7.75 (s, 1H, $H^{b}$), 7.50-7.48 (d, 4H, *J*= 12 Hz, B-Ph-$H^{h}$), 7.32-7.30 (t, 2H, *J*= 6 Hz, A-tpy-$H^{5''}$), 7.16-7.15 (d, 4H, *J*= 6 Hz, B-tpy-$H^{5,5''}$), 7.01-6.99 (d, 4H, *J*= 12 Hz, A-Ph-$H^{j}$), 3.86 (s, 6H, $H^{c}$), 2.50 (s, 12H, $H^{d}$). $^{13}$C NMR (151 MHz, $CDCl_3$) δ 160.44, 156.39, 156.21, 156.01, 155.82, 155.43, 154.87, 149.74, 149.63, 149.53, 148.99, 148.03, 140.79, 140.48, 138.00, 137.41, 136.87, 136.72, 136.26, 130.81, 130.51, 128.57, 127.50, 124.83, 123.71, 122.10, 121.53, 120.71, 118.96, 118.45, 118.27, 114.27, 21.37. ESI-MS (1423.7 calcd. For $C_{96}H_{70}N_{12}O_2$): *m/z* 1425.501 $[M + H^+]^+$ (calcd *m/z*: 1425.501).

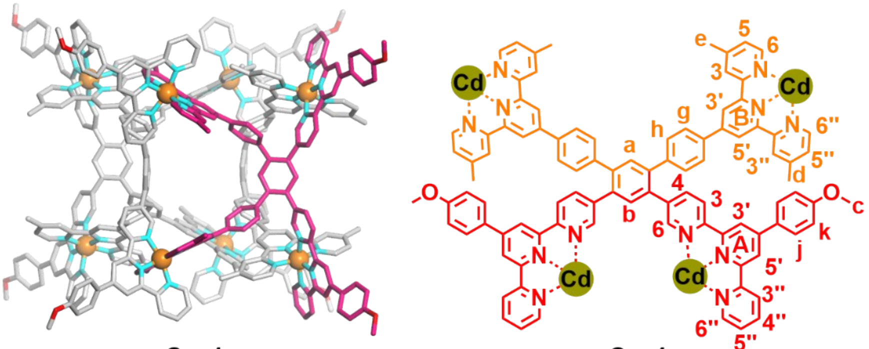


$C_{2h}$-**1** $C_{2h}$-**1**

**Cube-shaped supermolecule $C_{2h}$-1:** Ligand **L** (30.0 mg, 21.1 μmol), and $Cd(NO_3)_2$ (13.1 mg, 42.2 μmol) was added in a 100 mL flask, then a solvent mixture of $CHCl_3$/MeOH (45 mL, v/v, 3/4) was added. The mixture was refluxed for 12 h, after cooled to ambient temperature, excess $LiNTf_2$ in MeOH was added to get a white precipitate, which was filtered and washed with $H_2O$ and MeOH to generate a white solid: 60.1 mg (96%). $^1$H NMR (400 MHz, $CD_3CN$) δ 9.49 (s, 8H, B-tpy-$H^{3'}$), 9.32 (s, 8H, B-tpy-$H^{3}$), 8.92-8.90 (m, 16H, A-tpy-$H^{3'}$, A-tpy-$H^{3''}$), 8.68 (s, 8H, A-tpy-$H^{5'}$), 8.57-8.56 (d, 8H, *J*= 4 Hz, A-tpy-$H^{6''}$), 8.43-8.36 (m, 24H, A-tpy-$H^{4''}$, A-tpy-$H^{3}$, B-tpy-$H^{5'}$), 8.25-8.22 (m, 24H, B-tpy-$H^{3''}$, B-Ph-$H^{g}$), 8.10-8.08 (d, 16H, *J*= 8 Hz, A-Ph-$H^{j}$), 7.95-7.93 (d, 8H, *J*= 12 Hz, A-tpy-$H^{4}$), 7.86-7.85 (d, 8H, *J*= 4 Hz, B-tpy-$H^{6}$), 7.82-7.81 (d, 8H, *J*= 4 Hz, B-tpy-$H^{6''}$), 7.76-7.73 (t, 6H, A-tpy-$H^{5''}$),

7.27-7.25 (d, 8H, $J$= 8 Hz, B-tpy-$H^{5}$), 7.23-7.21 (d, 16H, $J$= 4 Hz, A-Ph-$H^{k}$), 7.08-7.06 (d, 16H, $J$= 8 Hz, B-Ph-$H^{h}$), 7.04-7.03 (d, 8H, $J$= 4 Hz, B-tpy-$H^{5''}$), 6.99 (s, 4H, $H^{a}$), 6.85 (s, 4H, $H^{b}$), 5.93 (s, 8H, A-tpy-$H^{6}$), 5.93 (s, 24H, $H^{c}$), 2.45 (s, 24H, $H^{e}$), 2.42 (s, 24H, $H^{d}$). ESI-MS (10640.0 calcd. For $C_{412}H_{280}Cd_{8}F_{84}N_{64}O_{70}S_{28}$): $m/z$ 827.413 [M – $10NTf_2^{-}$]$^{10+}$ (calcd $m/z$: 827.413), 950.667 [M – $9NTf_2^{-}$]$^{9+}$ (calcd $m/z$: 950.667), 1104.362 [M – $8NTf_2^{-}$]$^{8+}$ (calcd $m/z$: 1104.362), 1302.253 [M – $7NTf_2^{-}$]$^{7+}$ (calcd $m/z$: 1302.253).

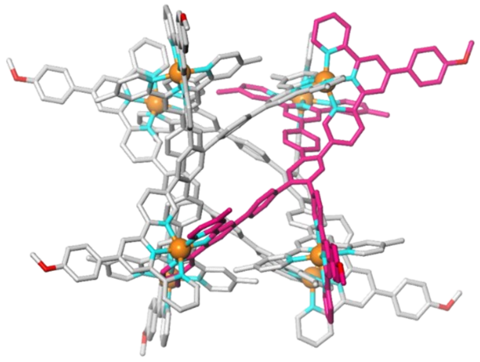

$D_2$-**2**

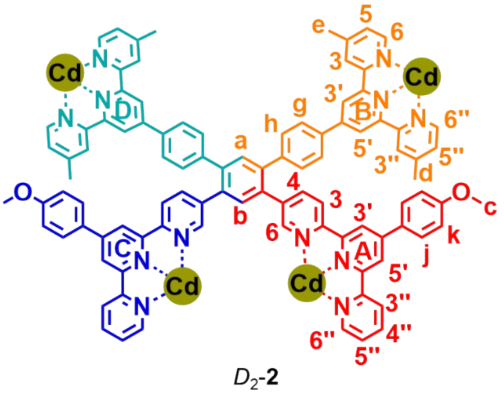


$D_2$-**2**

**Helical supermolecule $D_2$-2:** Weight 2 mg $C_{2h}$-**1** and dissolve it in a nuclear magnetic tube with deuterated acetonitrile, heated at 65 °C, and the first day was measured every 3 hours for nuclear magnetic resonance, and the data showed that there was no obvious change. Then the test was carried out at an interval of one day, and there was still no change after a week. So, the test was carried out at an interval of one week. After that a significant change was observed during the test in the third week, and it was changed to a daily test. Finally, all the transformations were detected in the fourth week, and there will be no changes in the following days. $^{1}$H NMR (500 MHz, $CD_3CN$) δ 9.12 (s, 4H, C-tpy-$H^{5'}$), 9.01-8.99 (m, 8H, A-tpy-$H^{5'}$, D-tpy-$H^{5'}$), 8.97-8.95 (m, 8H, C-tpy-$H^{3'}$, D-tpy-$H^{3'}$), 8.82 (m, 8H, A-tpy-$H^{3''}$, C-tpy-$H^{3''}$), 8.73 (m, 8H, B-tpy-$H^{3'}$, B-tpy-$H^{5'}$), 8.71-8.68 (d, 4H, $J$= 15 Hz, C-tpy-$H^{3}$), 8.66 (s, 4H, A-tpy-$H^{3'}$), 8.56 (m, 8H, B-tpy-$H^{3}$, B-tpy-$H^{3''}$), 8.49 (m, 4H, A-tpy-$H^{3}$), 8.44 (m, 8H, D-tpy-$H^{3}$, D-tpy-$H^{3''}$), 8.29-8.28 (m, 12H, C-tpy-$H^{4}$, C-Ph-$H^{j}$), 8.20 (s, 4H, $H^{a}$), 8.17-8.16 (m, 16H, A-tpy-$H^{6''}$, C-tpy-$H^{6''}$, A-Ph-$H^{j}$), 8.09-8.08 (d, 8H, $J$= 5 Hz, B-tpy-$H^{6}$, B-tpy-$H^{6''}$), 7.88-7.82 (m, 20H, A-tpy-$H^{4''}$, B-Ph-$H^{g}$, D-Ph-$H^{g}$), 7.77-7.75 (m, 8H, A-tpy-$H^{4}$, C-tpy-$H^{4''}$), 7.69 (s, 4H, $H^{b}$), 7.54-7.53 (m, 4H, C-tpy-$H^{6}$), 7.50-7.48 (m, 8H, D-tpy-$H^{6}$, D-tpy-$H^{6''}$), 7.33-7.27 (m, 32H, A-tpy-$H^{5''}$, C-tpy-$H^{5''}$, B-Ph-$H^{h}$, C-Ph-$H^{k}$, D-Ph-$H^{h}$), 7.12-7.11 (m, 8H, D-tpy-$H^{5}$, D-tpy-$H^{5''}$), 7.06 (m, 4H, A-Ph-$H^{k}$), 6.93-6.92 (d, 8H, $J$= 5 Hz, B-tpy-$H^{5}$, B-tpy-$H^{5''}$), 5.80 (s, 4H, A-tpy-$H^{6}$), 3.99 (s, 12H, C-$H^{c}$), 3.94 (s, 12H, A-$H^{c}$). ESI-MS (11076.2 calcd. For $C_{416}H_{280}Cd_{8}F_{96}N_{64}O_{72}S_{32}$): $m/z$ 827.414 [M – $10NTf_2^{-}$]$^{10+}$ (calcd $m/z$: 827.414), 950.657 [M – $9NTf_2^{-}$]$^{9+}$ (calcd $m/z$: 950.657), 1104.361 [M – $8NTf_2^{-}$]$^{8+}$ (calcd $m/z$: 1104.361, 1302.249 [M – $7NTf_2^{-}$]$^{7+}$ (calcd $m/z$: 1302.249).

## 3. NMR and MS spectra data of Compound and supramolecule.

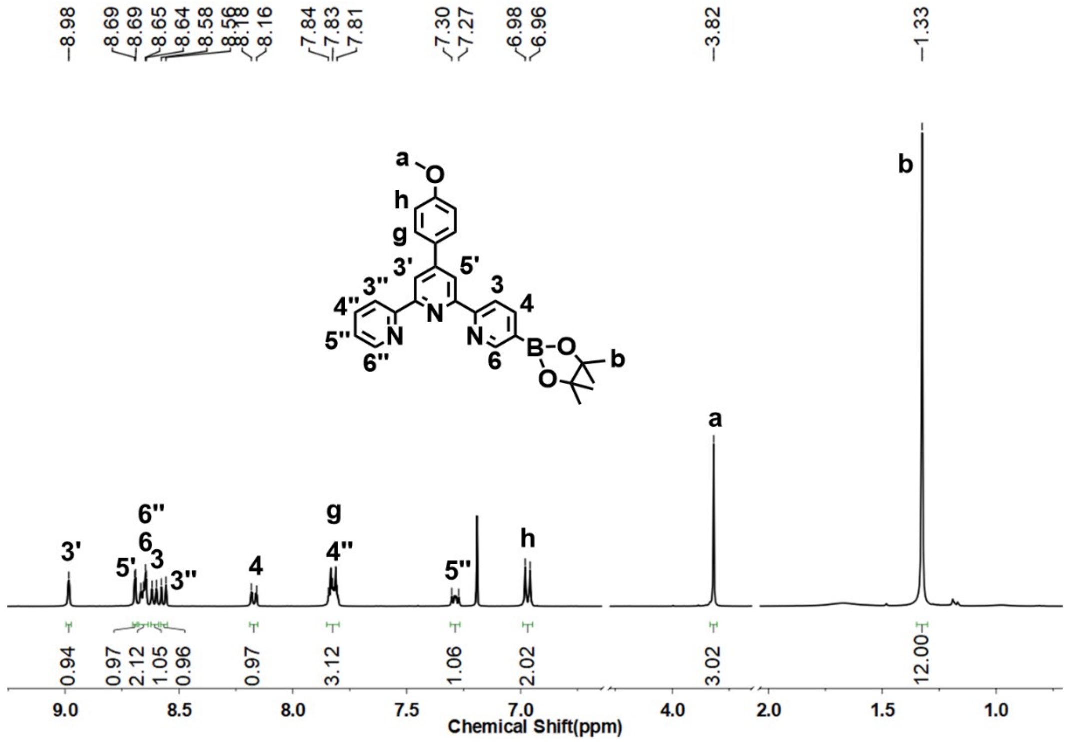

Figure S1: $^{1}H$ NMR spectrum (400 MHz, 298 K) of **2** in $CDCl_3$.

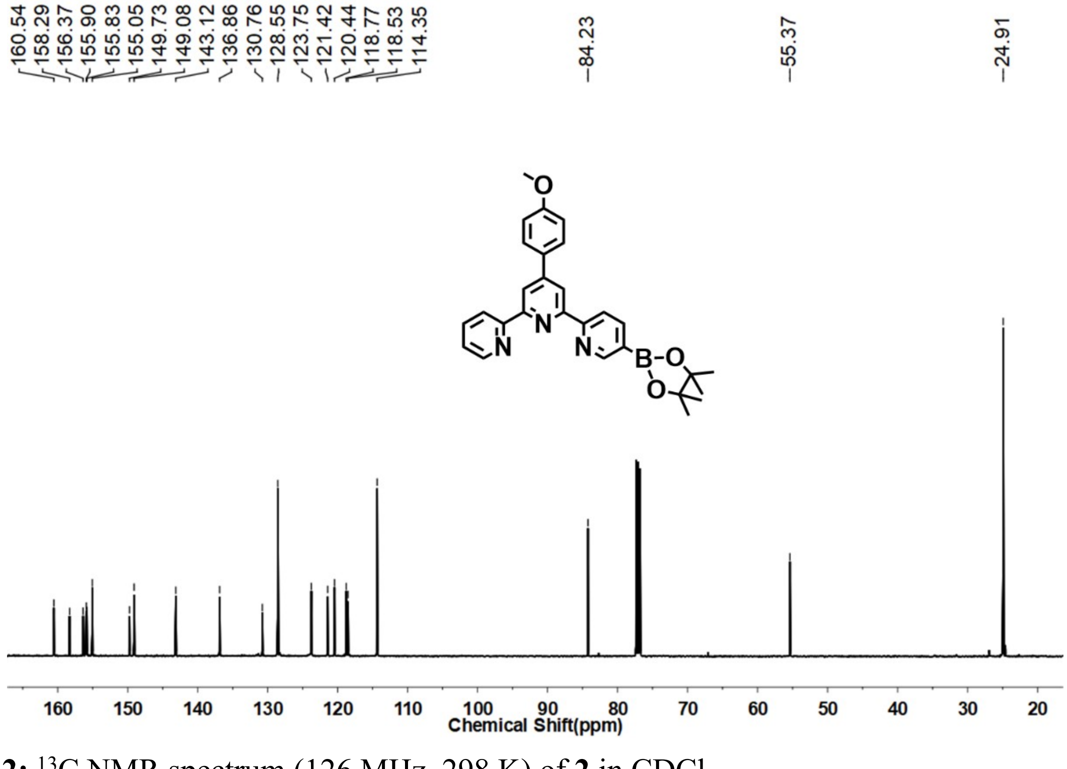

Figure S2: $^{13}C$ NMR spectrum (126 MHz, 298 K) of **2** in $CDCl_3$.

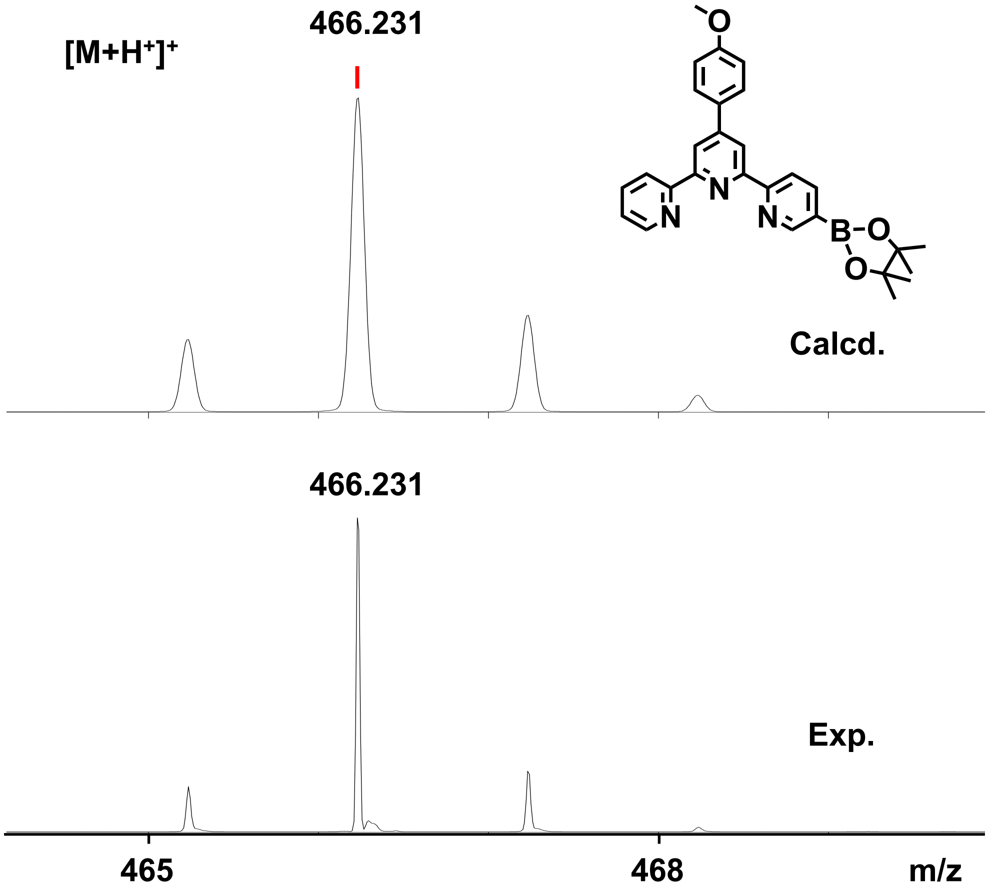


**Figure S3:** Isotope patterns spectrum of **2**.

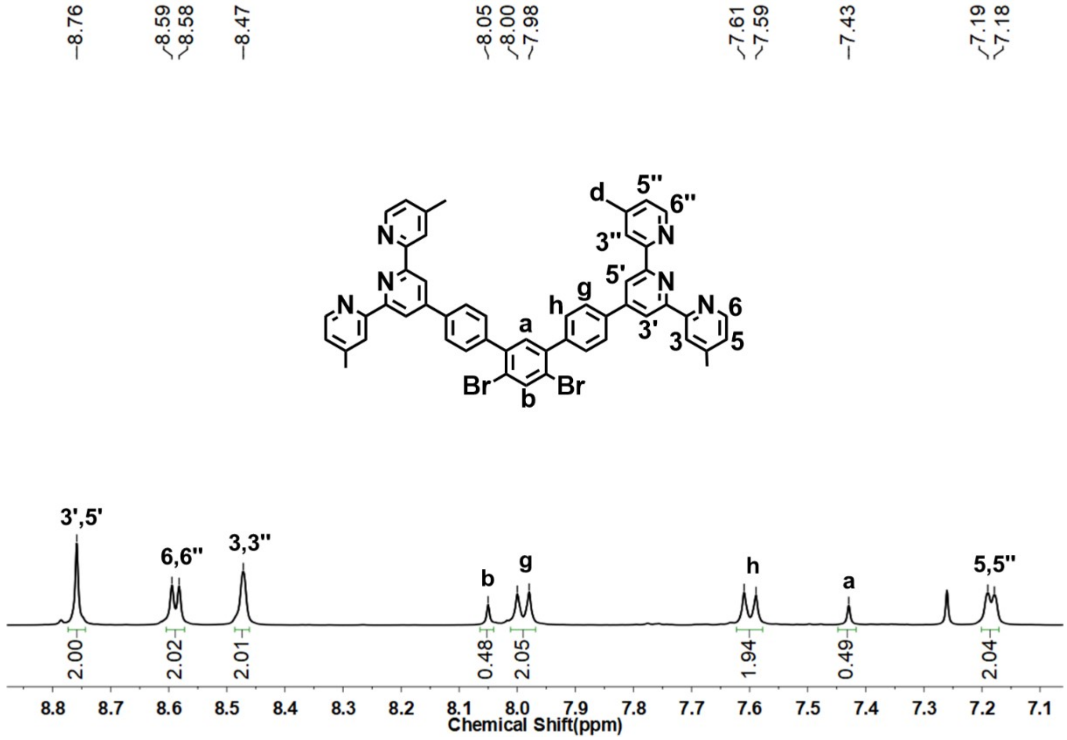


**Figure S4:** $^{1}$H NMR spectrum (400 MHz, 298 K) of **4** in $CDCl_3$.

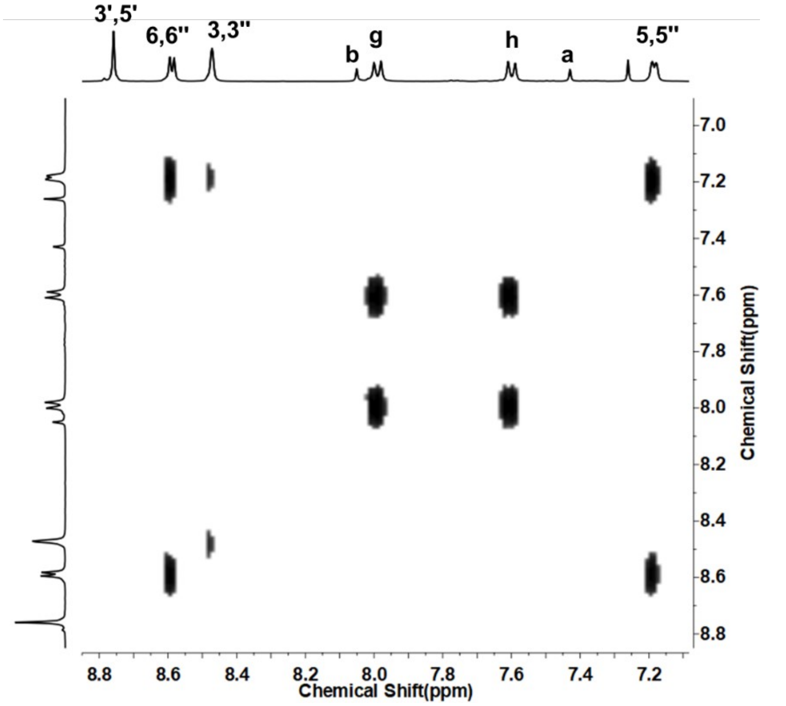


**Figure S5:** 2D COSY spectrum (400 MHz, 298 K) of **4** in $CDCl_3$.

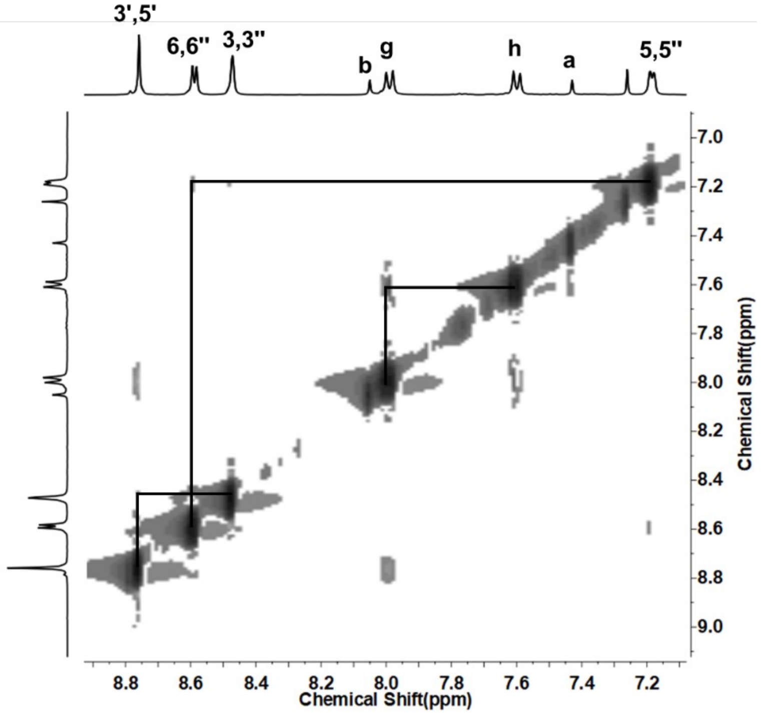


**Figure S6:** 2D NOESY spectrum (400 MHz, 298 K) of **4** in $CDCl_3$.

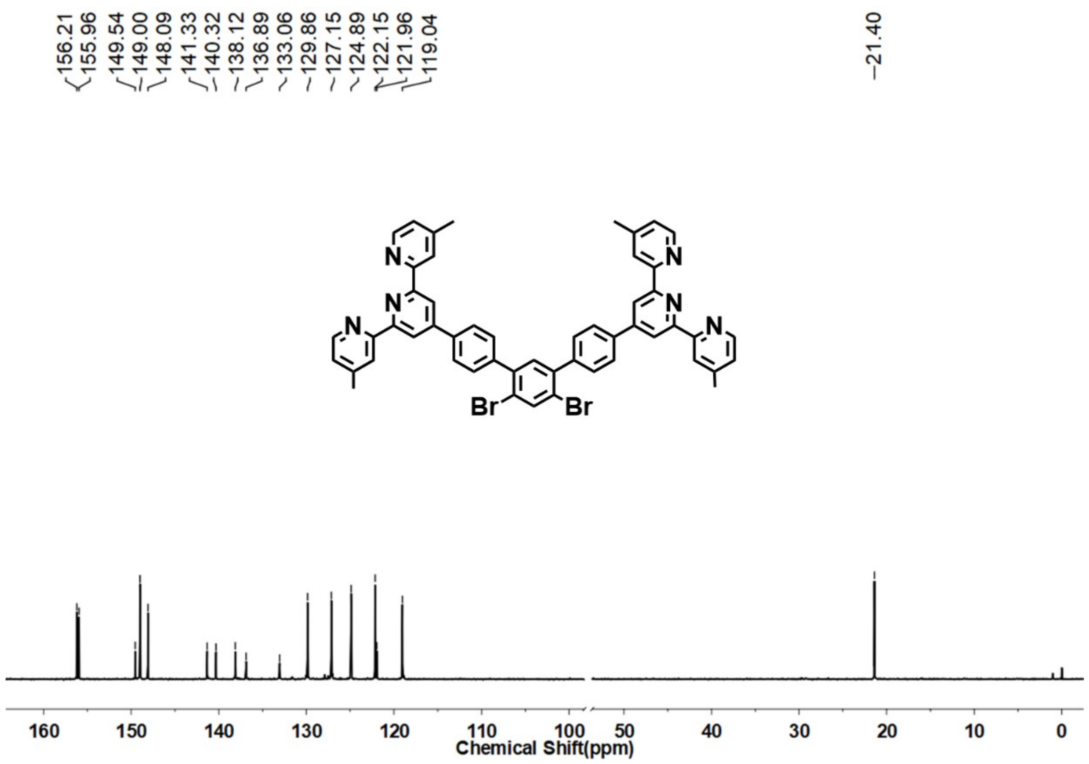


**Figure S7:** $^{13}C$ NMR spectrum (101 MHz, 298 K) of **4** in $CDCl_3$.

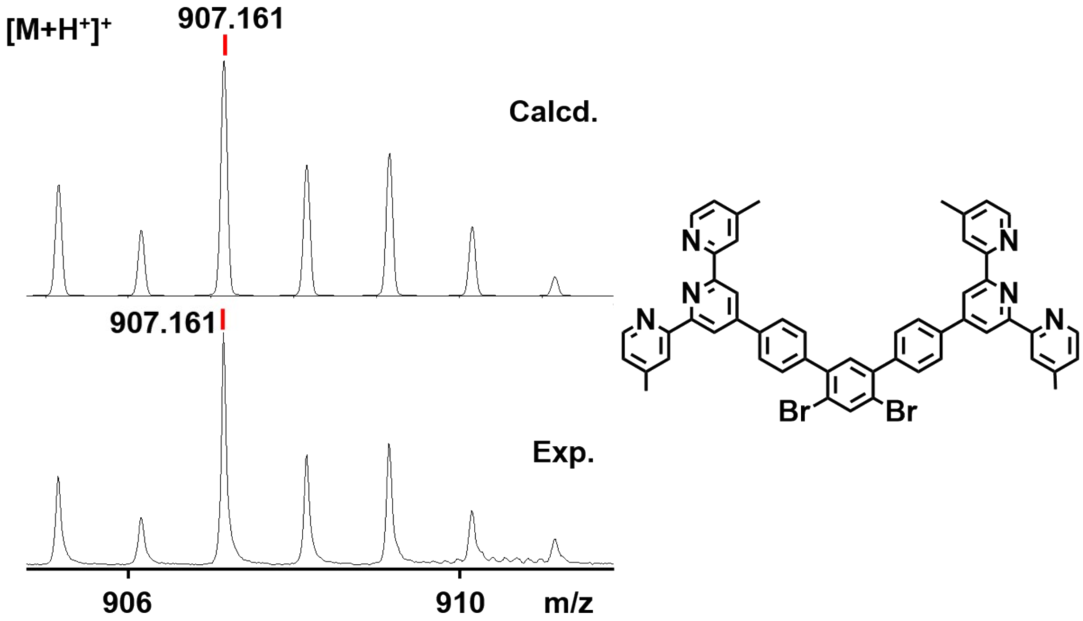


**Figure S8:** Isotope patterns spectrum of **4**.

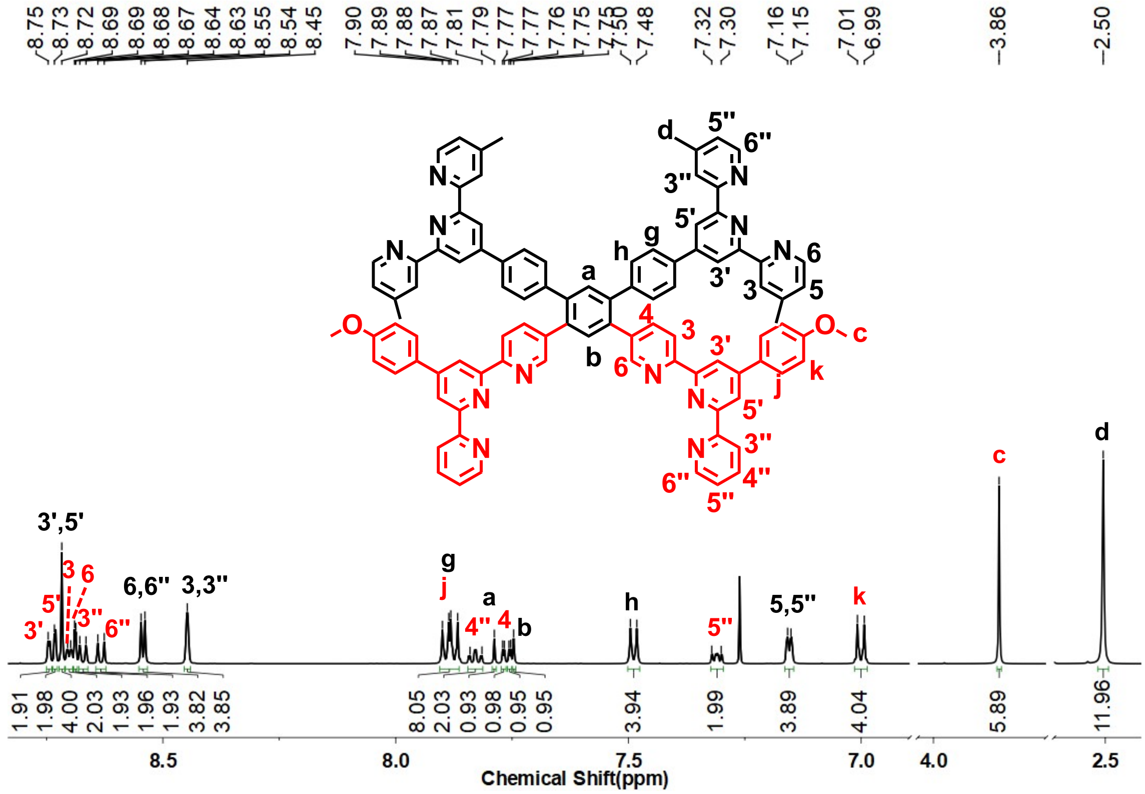


**Figure S9:** $^{1}H$ NMR spectrum (600 MHz, 298 K) of **L** in $CDCl_3$.

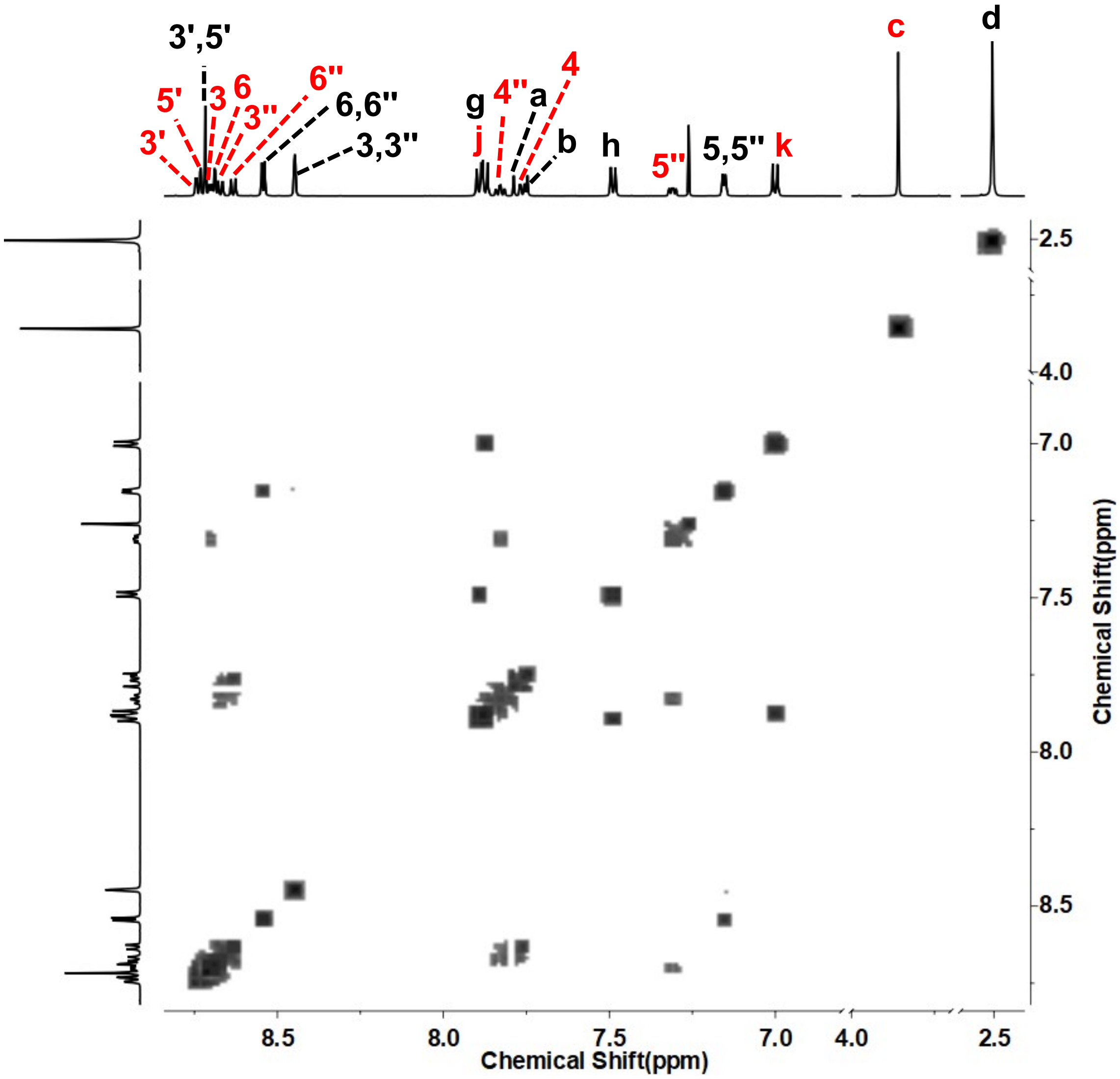


**Figure S10:** 2D COSY spectrum (600 MHz, 298 K) of **L** in $CDCl_3$.

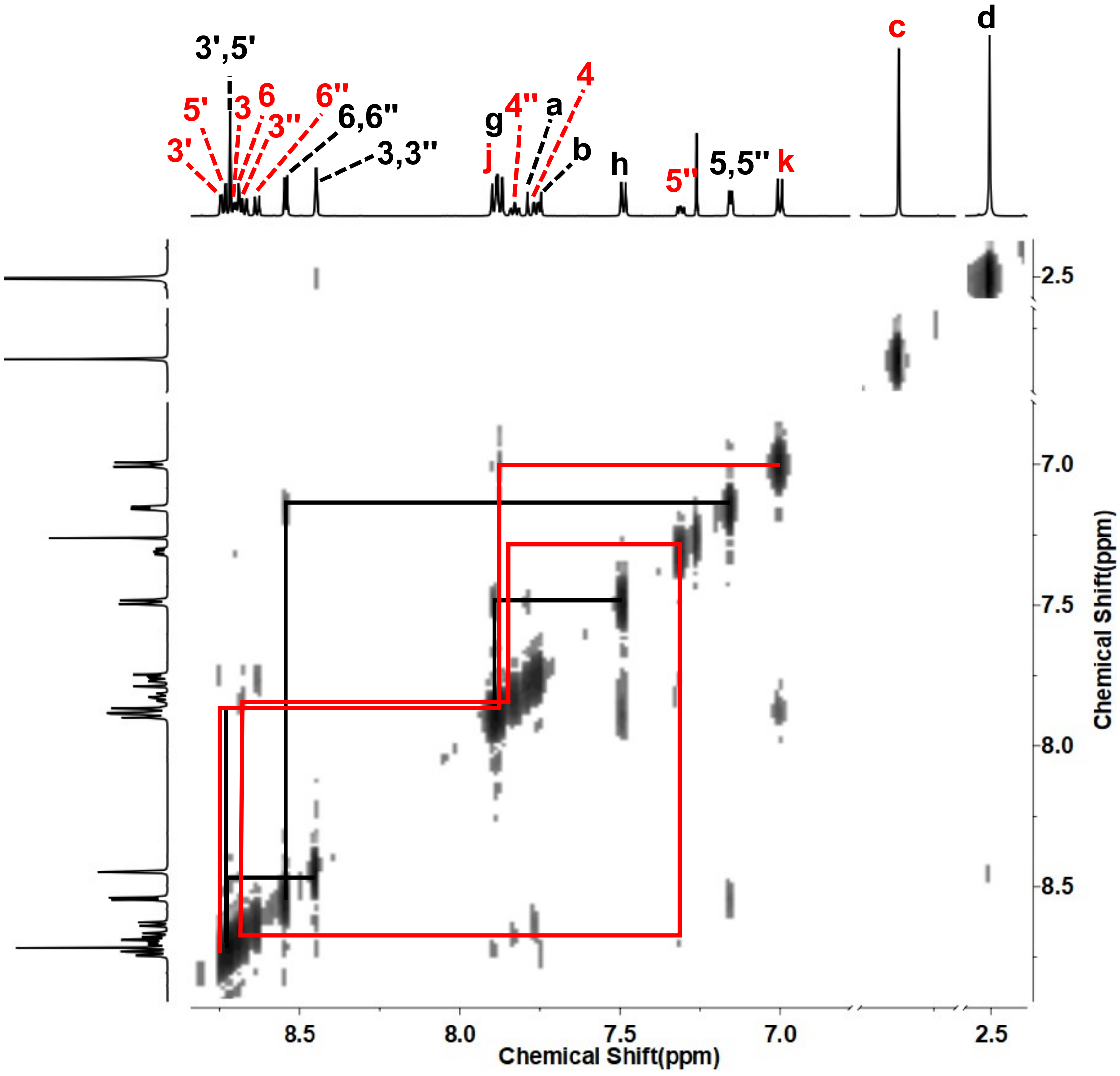


**Figure S11:** 2D NOESY spectrum (600 MHz, 298 K) of **L** in $CDCl_3$.

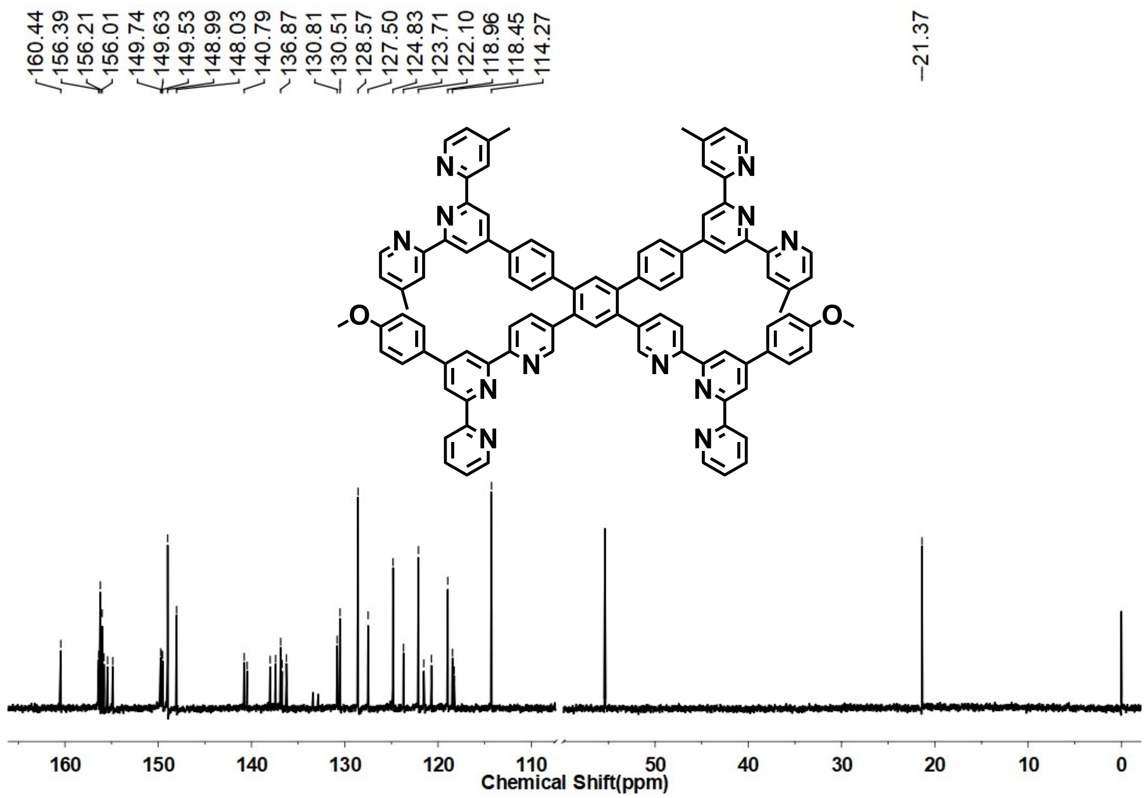


**Figure S12:** $^{13}C$ NMR spectrum (151 MHz, 298 K) of **L** in $CDCl_3$.

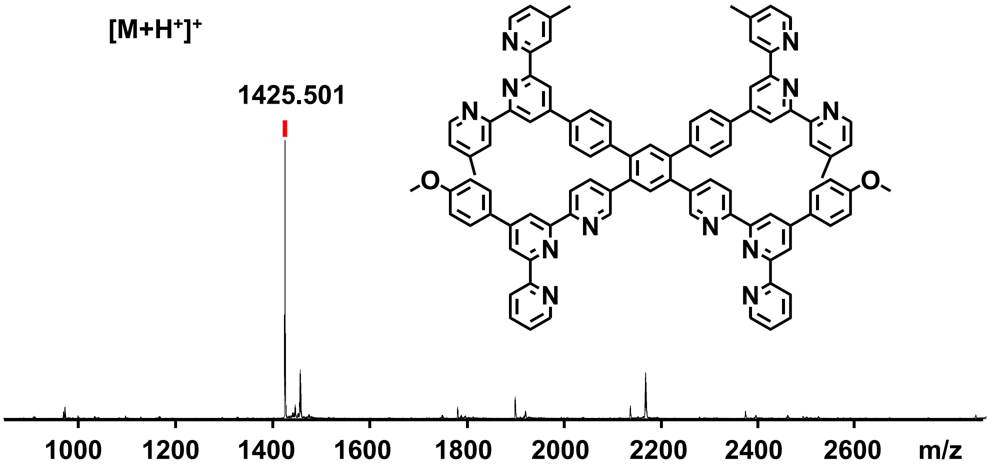


**Figure S13:** ESI-MS spectrum of **L**.

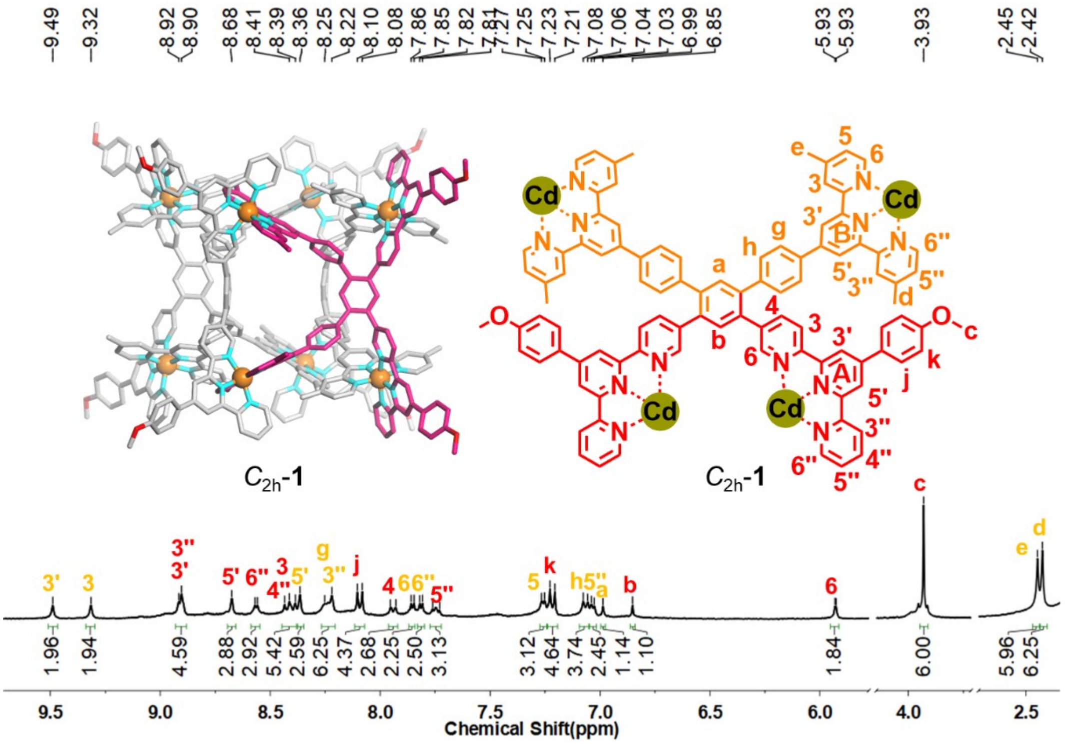


**Figure S14:** $^{1}H$ NMR spectrum (400 MHz, 298 K) of metallo-cube $C_{2h}$-**1** in $CD_3CN$.

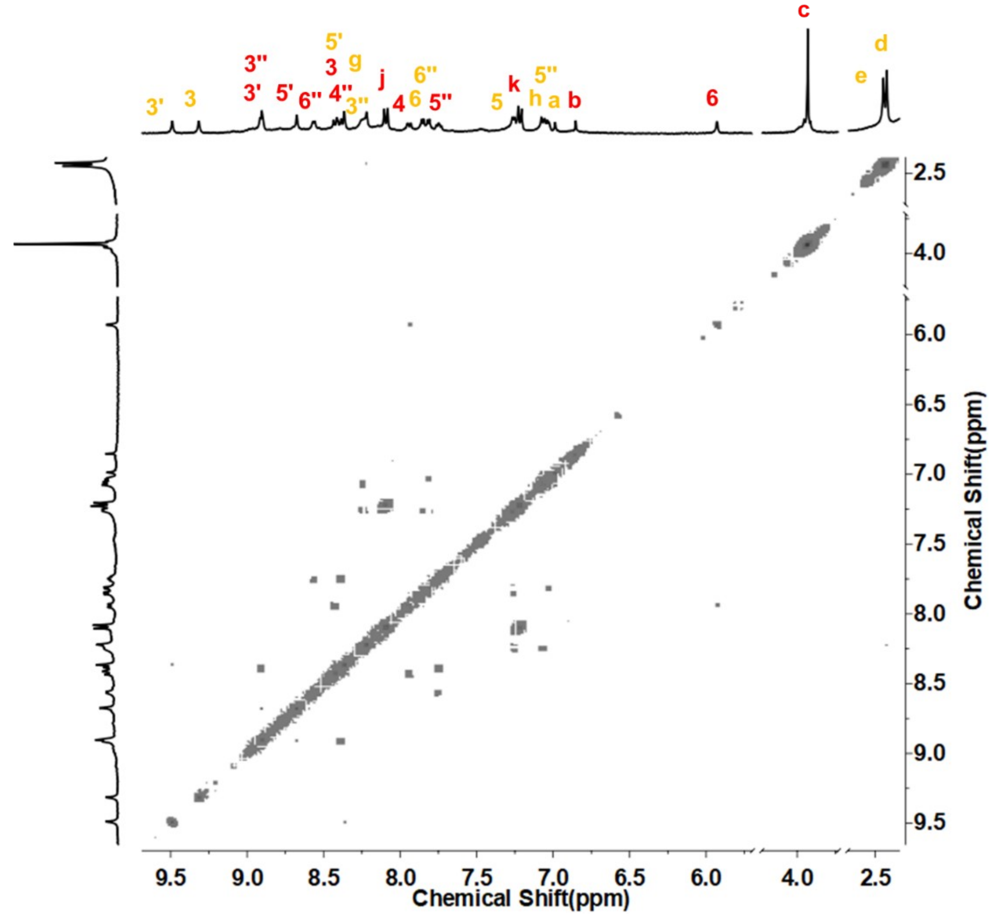


**Figure S15:** 2D COSY spectrum (400 MHz, 298 K) of metallo-cube $C_{2h}$-**1** in $CD_3CN$.

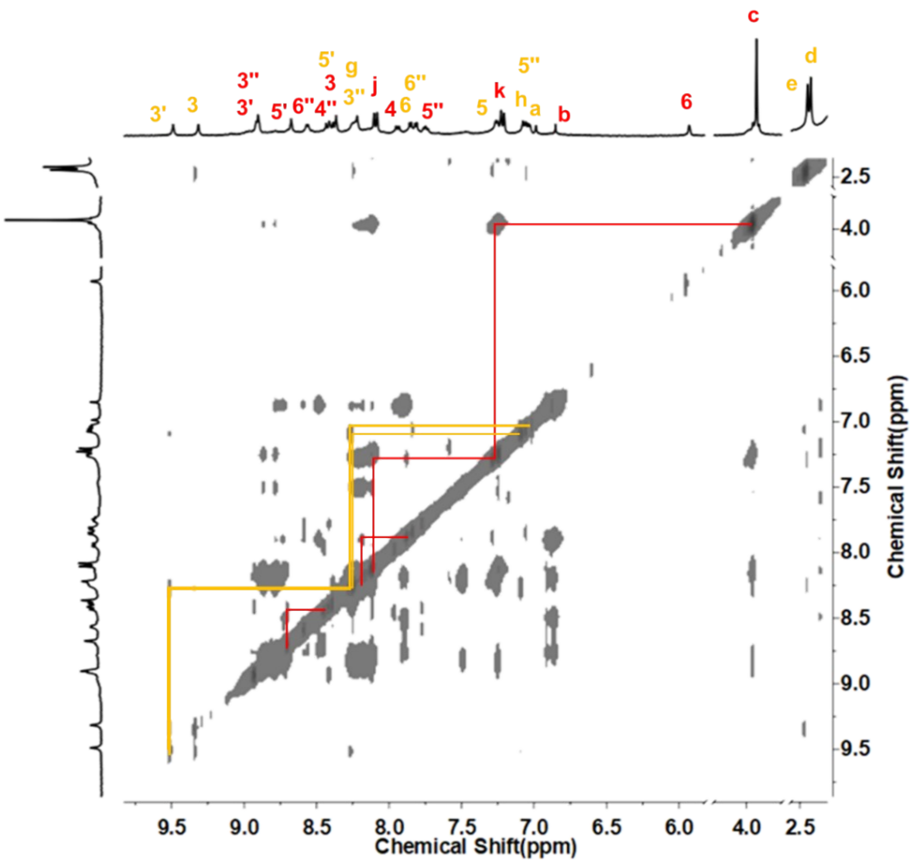


**Figure S16:** 2D NOESY spectrum (400 MHz, 298 K) of metallo-cube $C_{2h}$-**1** in $CD_3CN$.

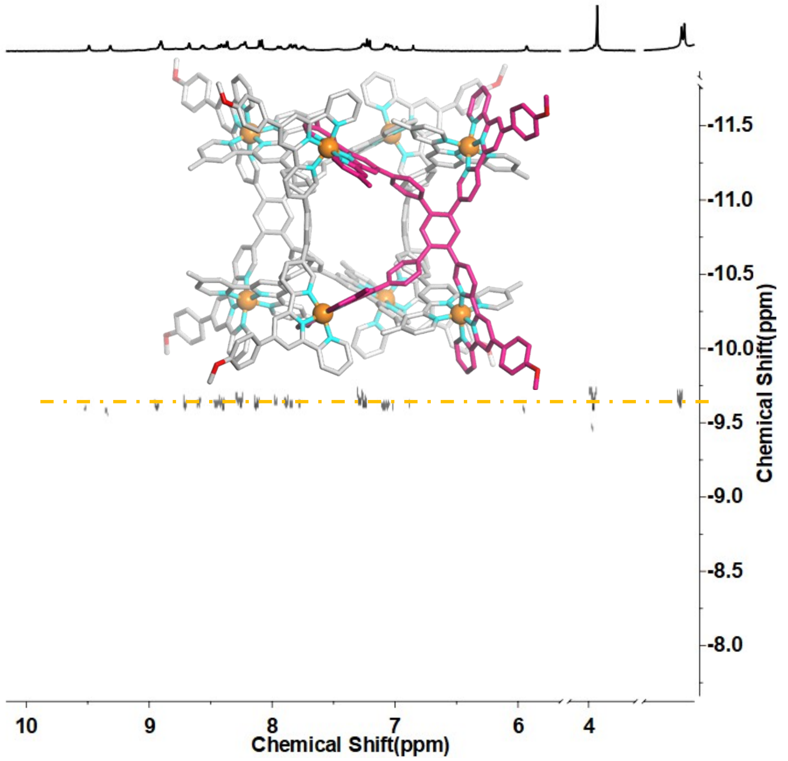


**Figure S17:** 2D DOSY spectrum (500 MHz, 298 K) of metallo-cube $C_{2h}$-**1** in $CD_3CN$.

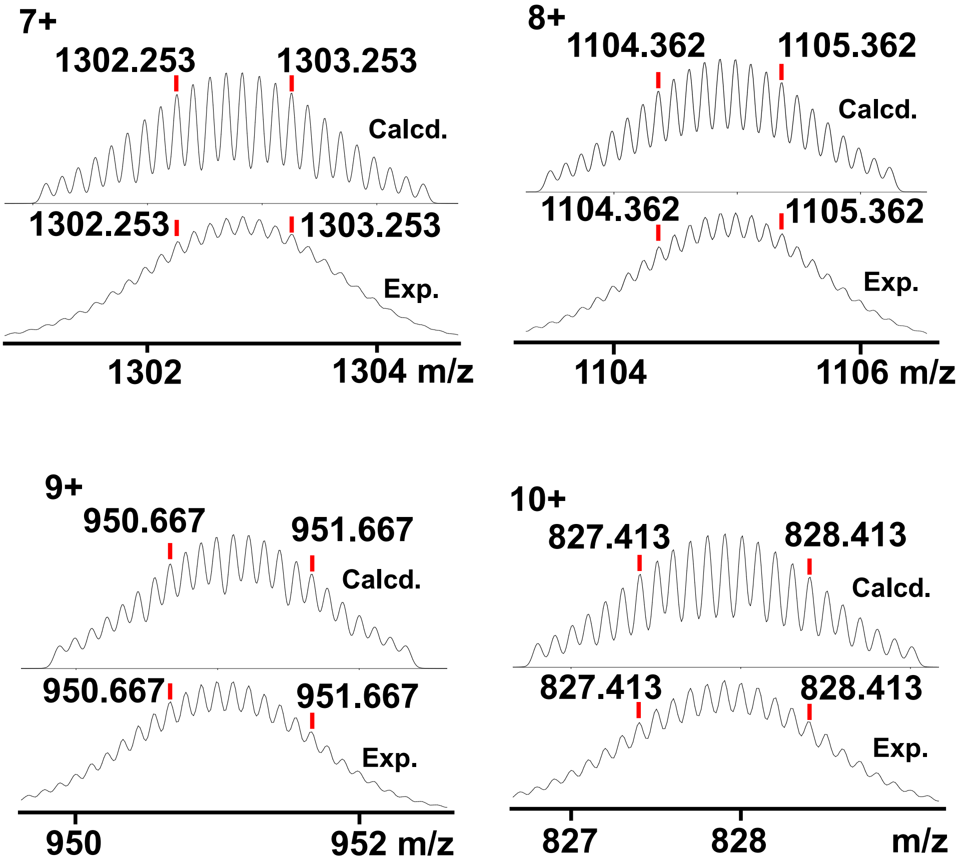


**Figure S18:** Isotope patterns spectrum of metallo-cube $C_{2h}$-**1** ($NTf_2^-$ as counterion).

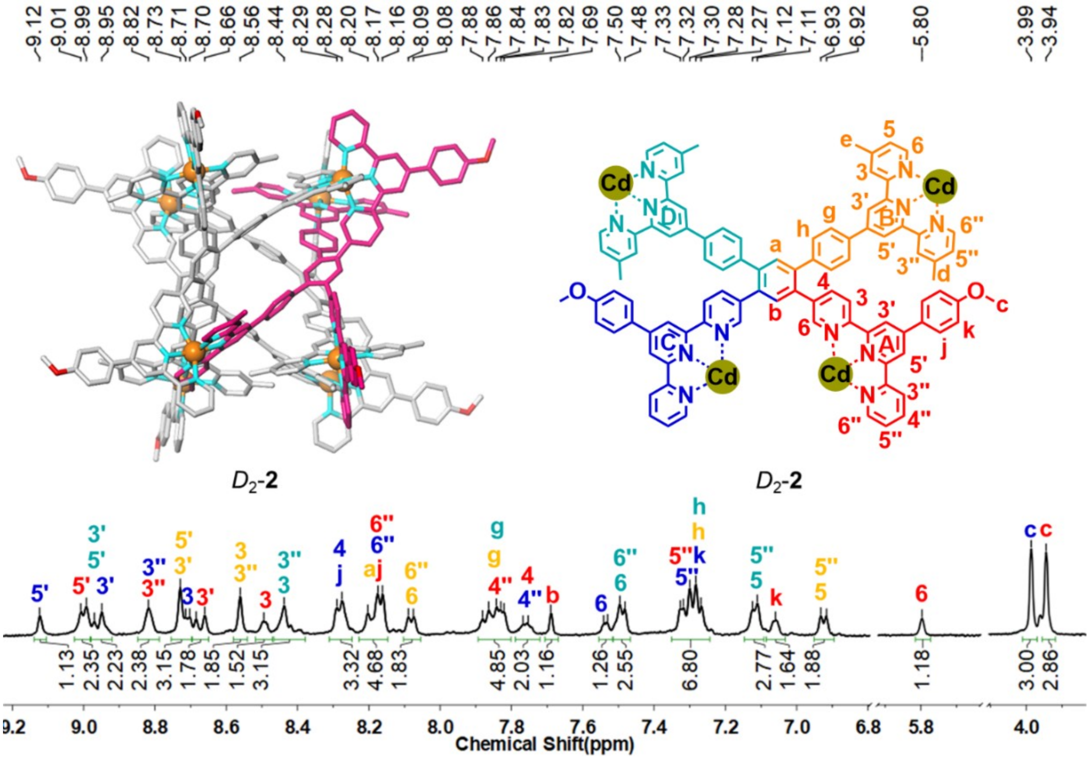


**Figure S19:** $^1$H NMR spectrum (400 MHz, 298 K) of metallo-cube $D_2$-**2** in $CD_3CN$.

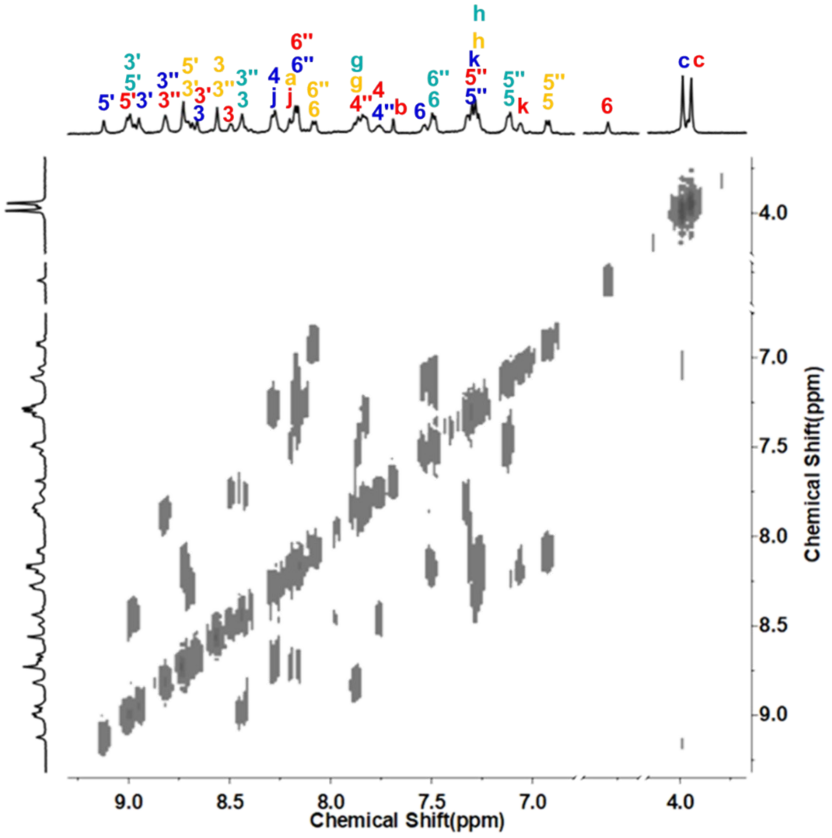


**Figure S20:** 2D COSY spectrum (400 MHz, 298 K) of metallo-cube $D_2$-**2** in $CD_3CN$.

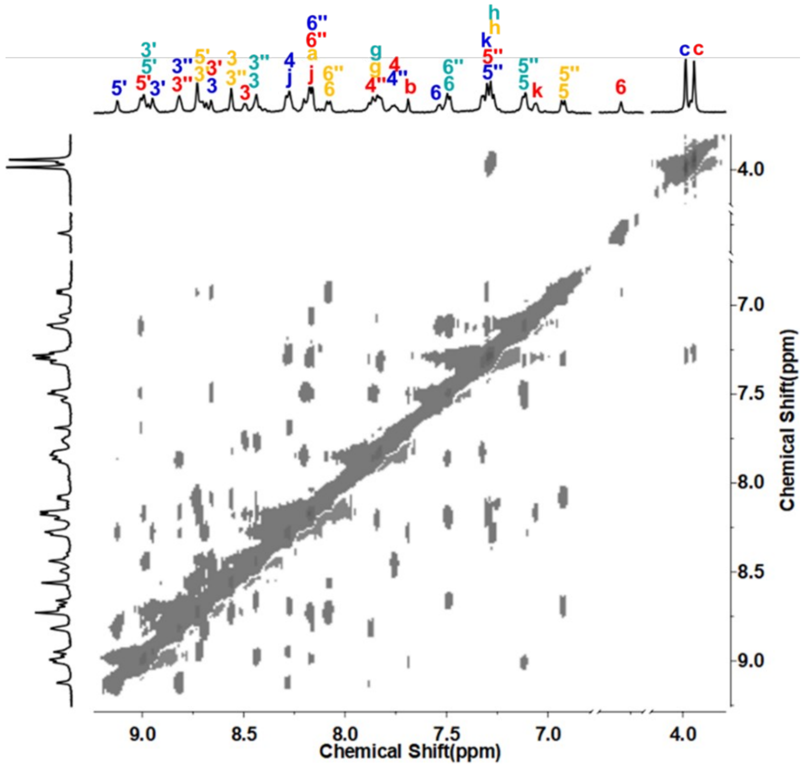


**Figure S21:** 2D NOESY spectrum (400 MHz, 298 K) of metallo-cube $D_2$-**2** in $CD_3CN$.

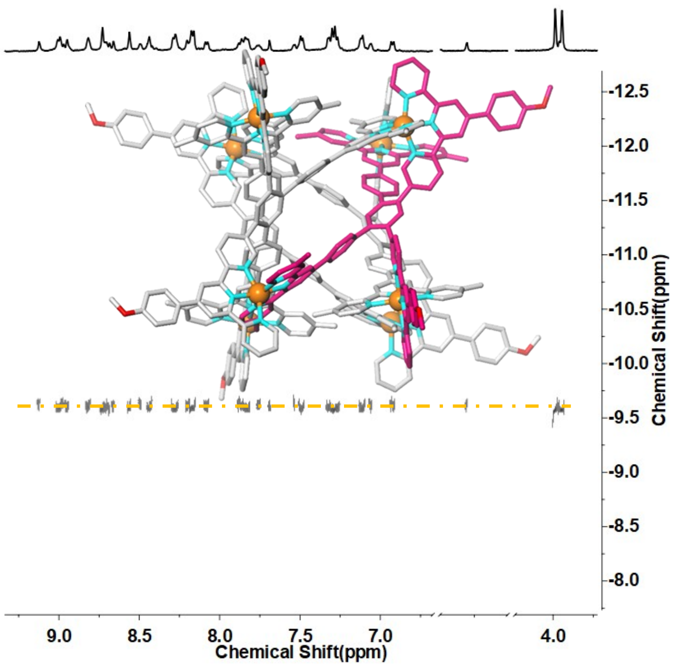


**Figure S22:** 2D DOSY spectrum (500 MHz, 298 K) of metallo-cube $D_2$-**2** in $CD_3CN$.

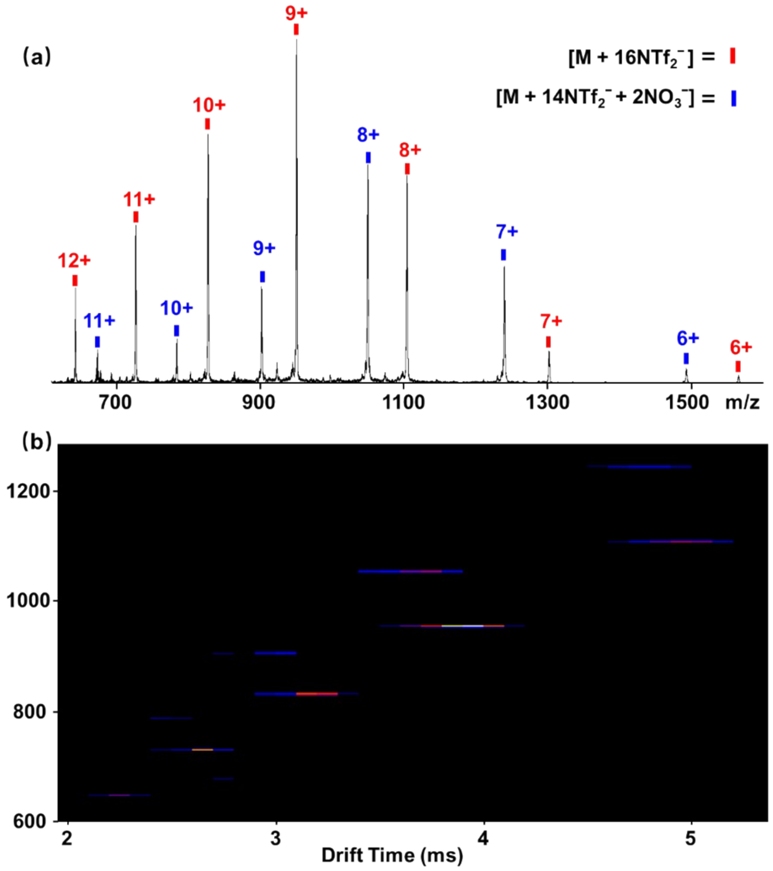


**Figure S23:** (a) ESI-MS spectrum and (b) ESI-TWIM-MS plot of metallo-cube $D_2$-**2** ($NTf_2^-$ and $NO_3^-$ as counterion).

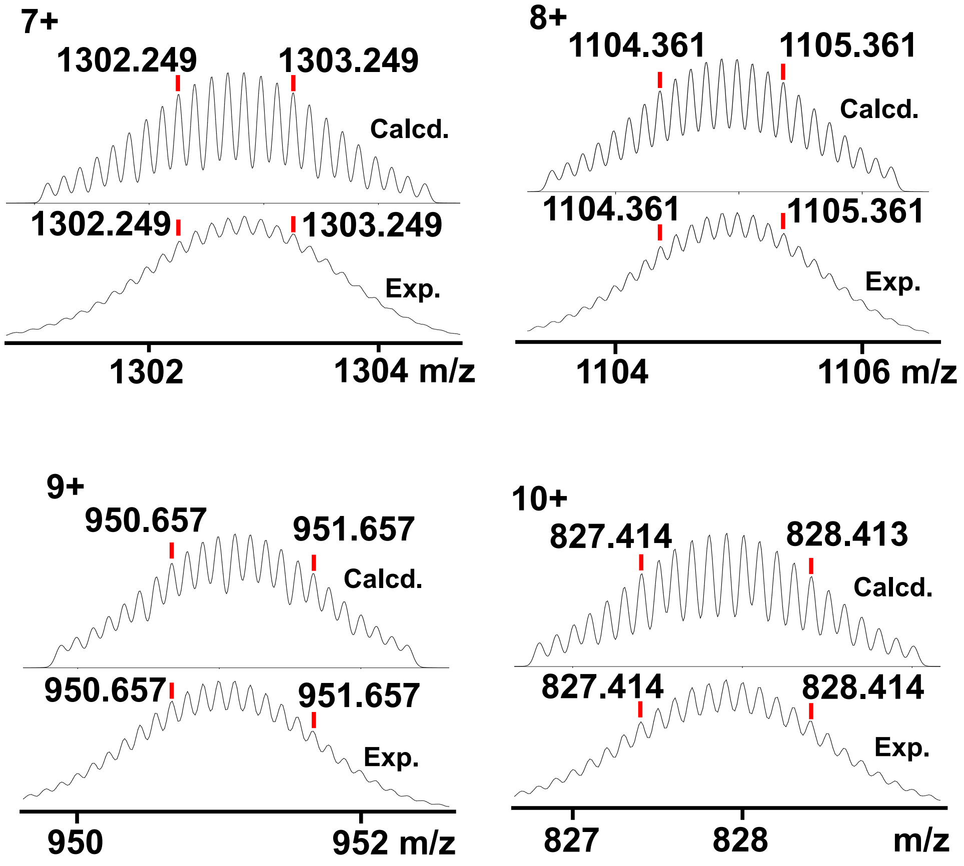


**Figure S24:** Isotope patterns spectrum of metallo-cube $D_2$-**2** ($NTf_2^-$ as counterion).

## 4. Host-guest complexation

4.1 neutral guests with no encapsulation

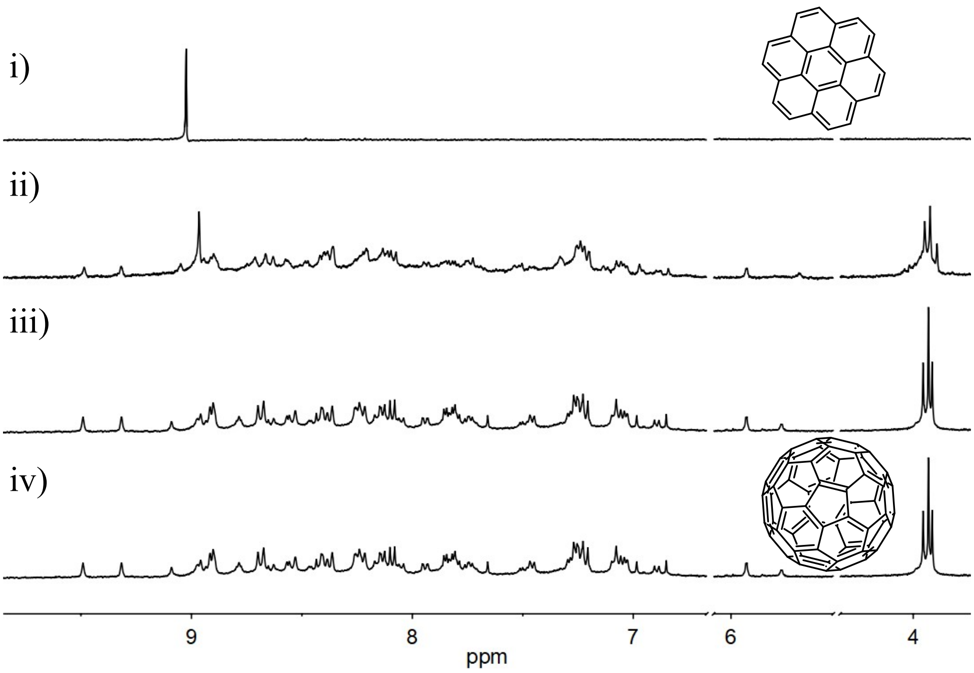


**Figure S25:** The comparison of $^1$H NMR spectra (500 MHz, 298 K, $CD_3CN$-$d_3$) of i) coronene (CO R); ii) COR + ($C_{2h}$-**1** + $D_2$-**2**); iii) $C_{2h}$-**1** + $D_2$-**2**; iv) $C_{60}$ + ($C_{2h}$-**1** + $D_2$-**2**). $C_{60}$ and COR cannot be enc apsulated by $C_{2h}$-**1** + $D_2$-**2**.

4.2 anionic guests can be encapsulated by host but don't cause any equilibrium shift between two conformers.

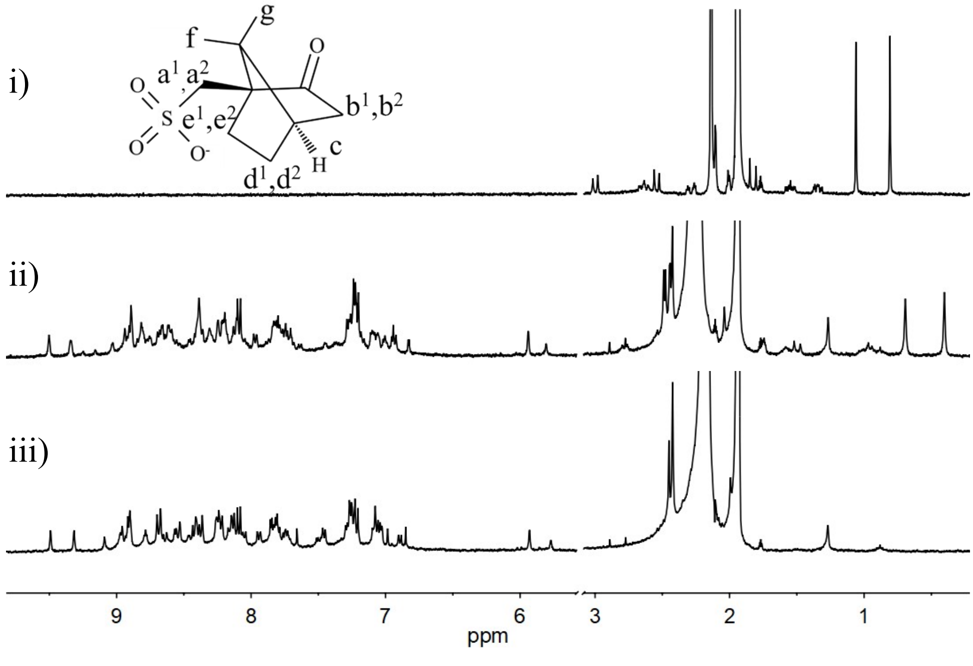


**Figure S26:** The comparison of full $^1$H NMR spectra (500 MHz, 298 K, $CD_3CN$-$d_3$) of i) *D*-sodium

camphor sulfonate; ii) *D*-sodium camphor sulfonate + ($C_{2h}$-**1** + $D_2$-**2**); iii) $C_{2h}$-**1**+ $D_2$-**2**. *D*-sodium camp hor sulfonate can be encapsulated by $C_{2h}$-**1**+ $D_2$-**2**.

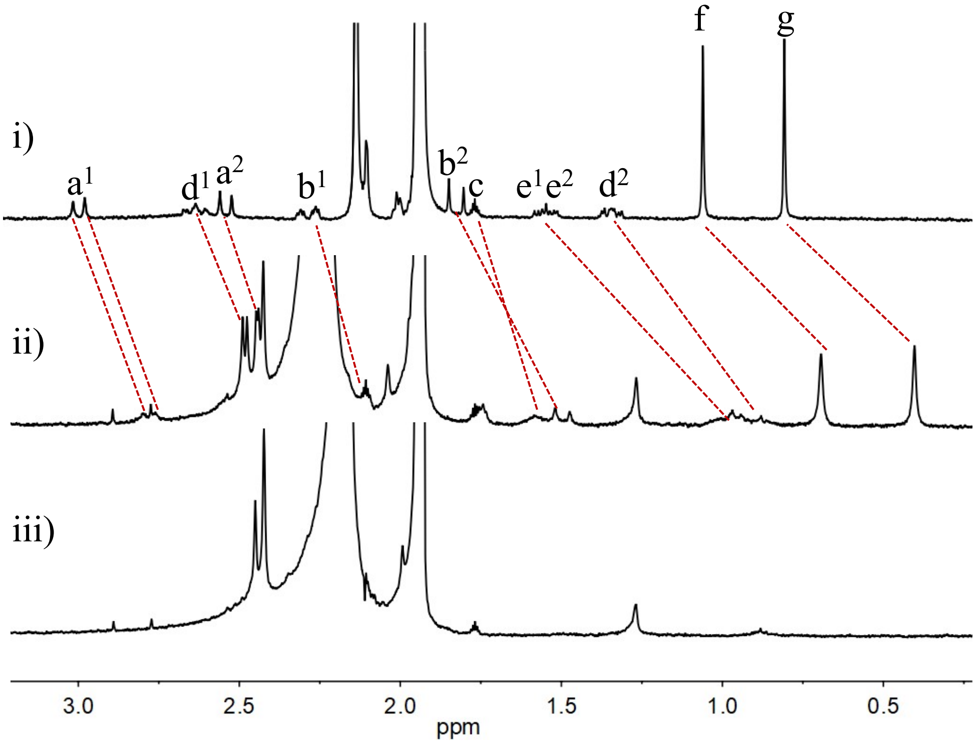


**Figure S27:** The comparison of enlarged $^1$H NMR spectra (500 MHz, 298 K, $CD_3CN$-$d_3$) of i) *D*-sodium camphor sulfonate; ii) *D*-sodium camphor sulfonate + ($C_{2h}$-**1** + $D_2$-**2**); iii) $C_{2h}$-**1**+ $D_2$-**2**. *D*-sodium camphor sulfonate can be encapsulated by $C_{2h}$-**1** + $D_2$-**2**.

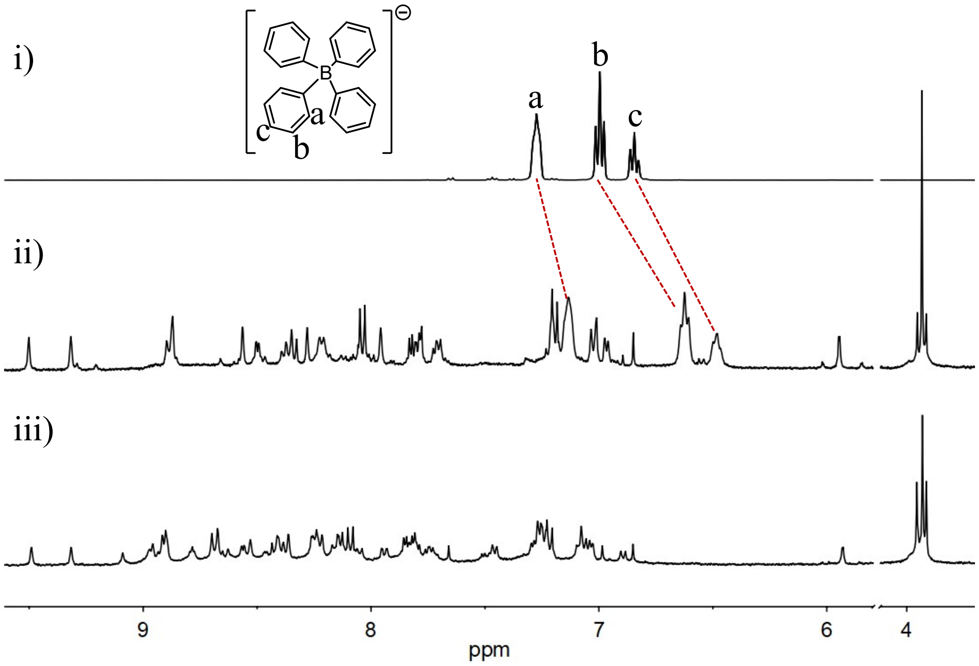


**Figure S28:** The comparison of $^1$H NMR spectra (500 MHz, 298 K, $CD_3CN$-$d_3$) of i) tetra-n-butylamm

onium tetraphenylborate; ii) tetra-n-butylammonium tetraphenylborate + ($C_{2h}$-**1** + $D_2$-**2**); iii) $C_{2h}$-**1**+ $D_2$ -**2**. Tetra-n-butylammonium tetraphenylborate can be encapsulated by $C_{2h}$-**1**+ $D_2$-**2**.

4.3 anionic guest can be encapsulated and causes equilibrium shift between the two conformers.

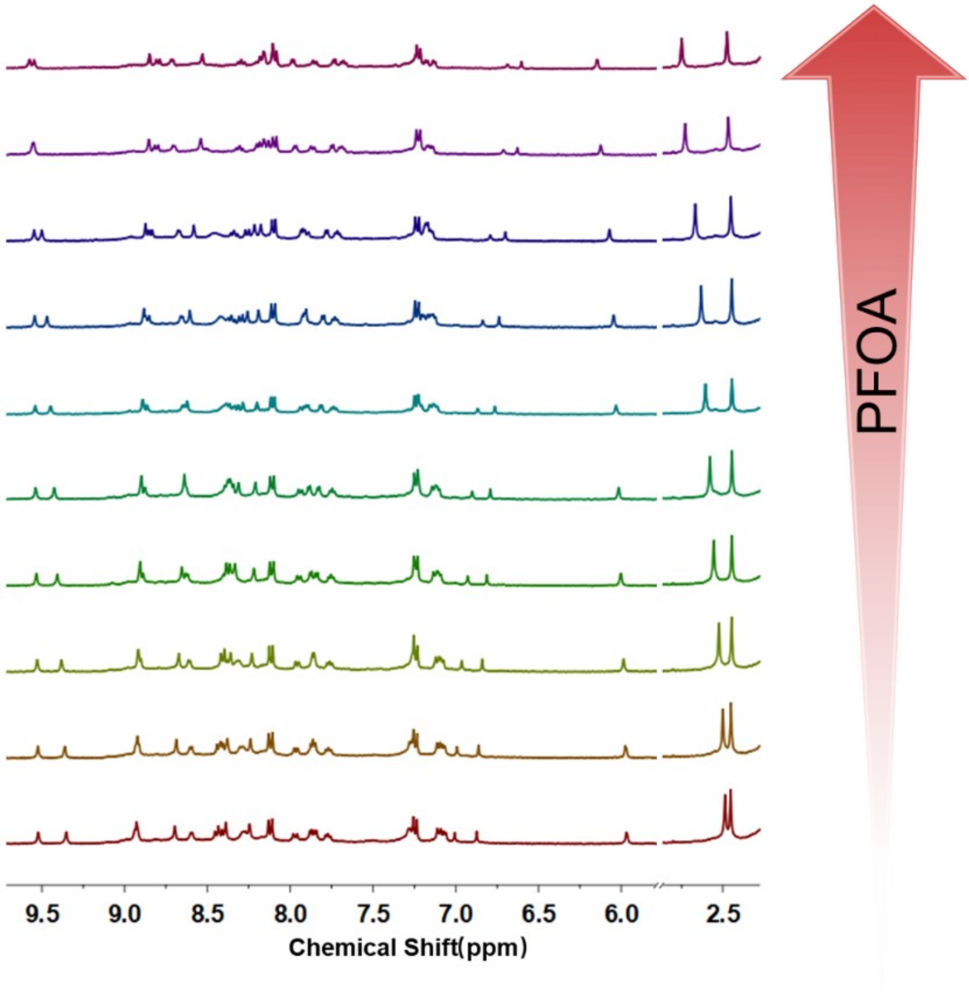


**Figure S29:** $^1$H NMR titration with the continuous addition of sodium perfluorooctanoate into the so lution of metallo-cage $C_{2h}$-**1** (sodium perfluorooctanoate concentration gradually increasing from bot tom to top).

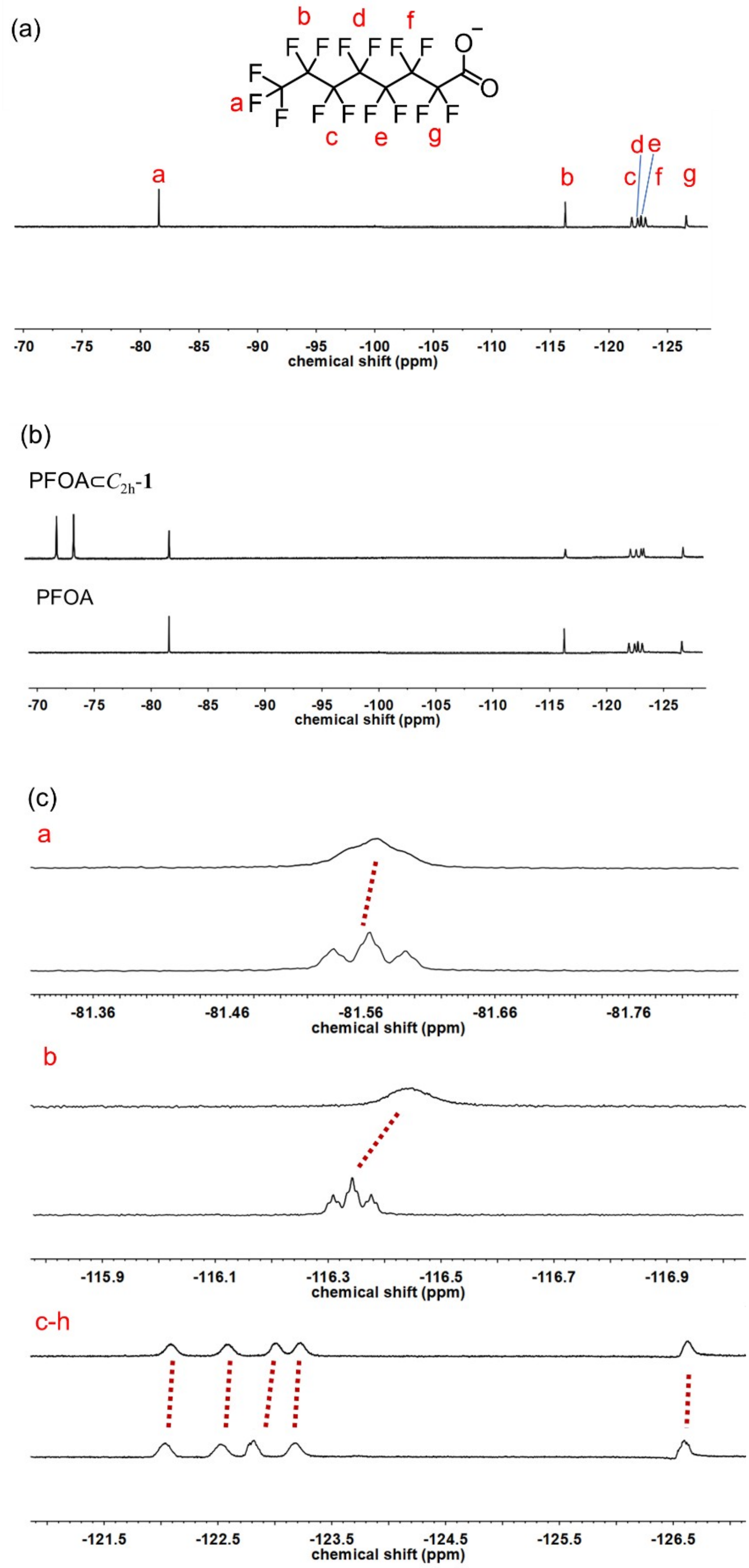


**Figure S30:** (a) $^{19}$F NMR (500 MHz, $CD_3CN$, 298 K) spectra of PFOA; (b) $^{19}$F NMR (500 MHz, $CD_3CN$, 298 K) spectra of the conformer $C_{2h}$-**1** and PFOA⊂ $C_{2h}$-**1**; (c) Partial $^{19}$F NMR (500 MHz, $CD_3CN$, 298 K) spectra of (b).

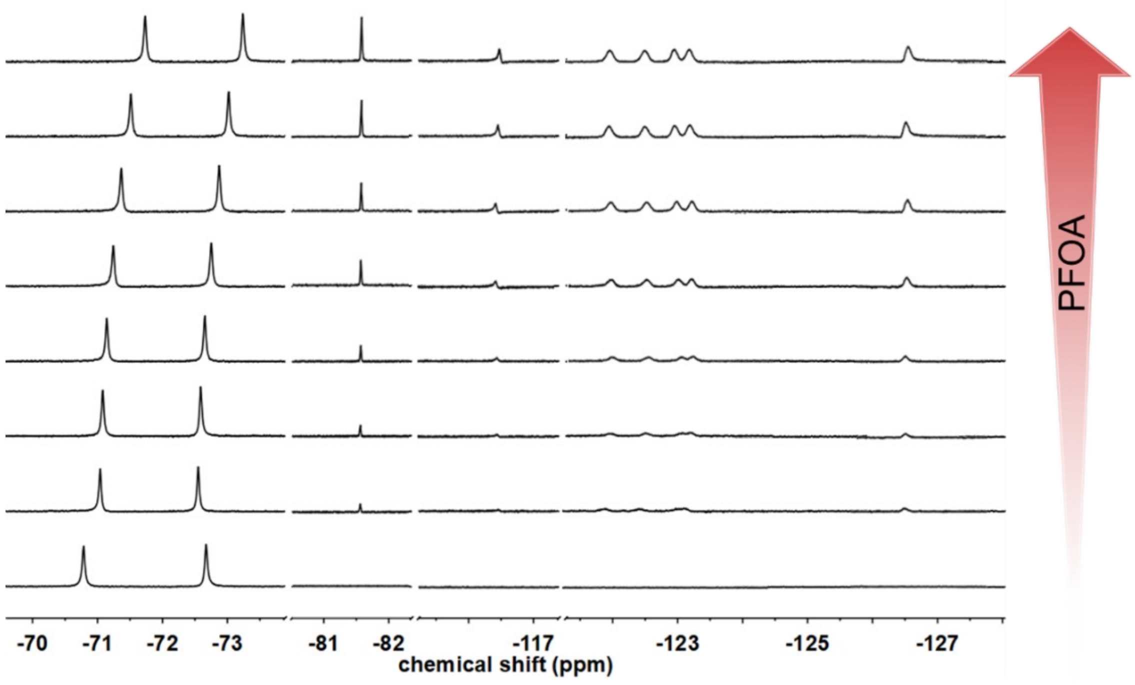


**Figure S31:** $^{19}$F NMR (500 MHz, $CD_3CN$, 298 K) titration with the continuous addition of sodium perfluorooctanoate into the solution of metallo-cage $C_{2h}$-**1** (sodium perfluorooctanoate concentration gradually increasing from bottom to top).

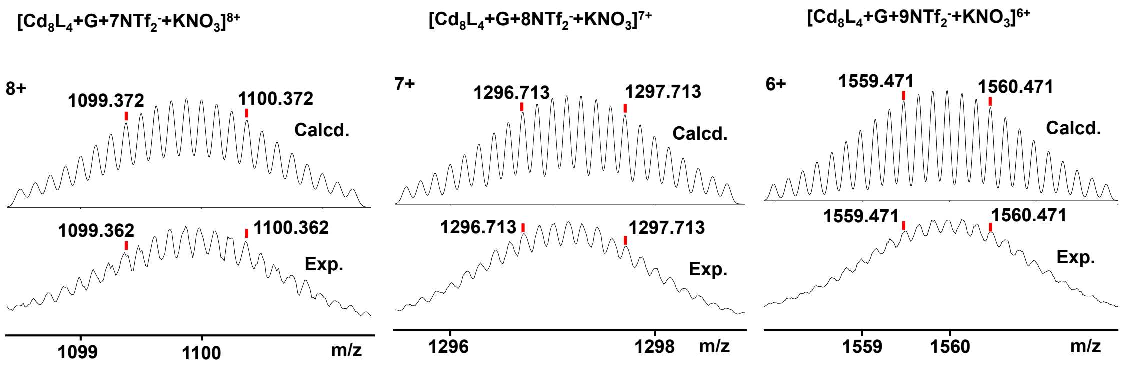


**Figure S32:** The isotopic patterns of three charge states (8+, 7+, 6+) in the host-guest complex PFOA⊂ $C_{2h}$-**1**.

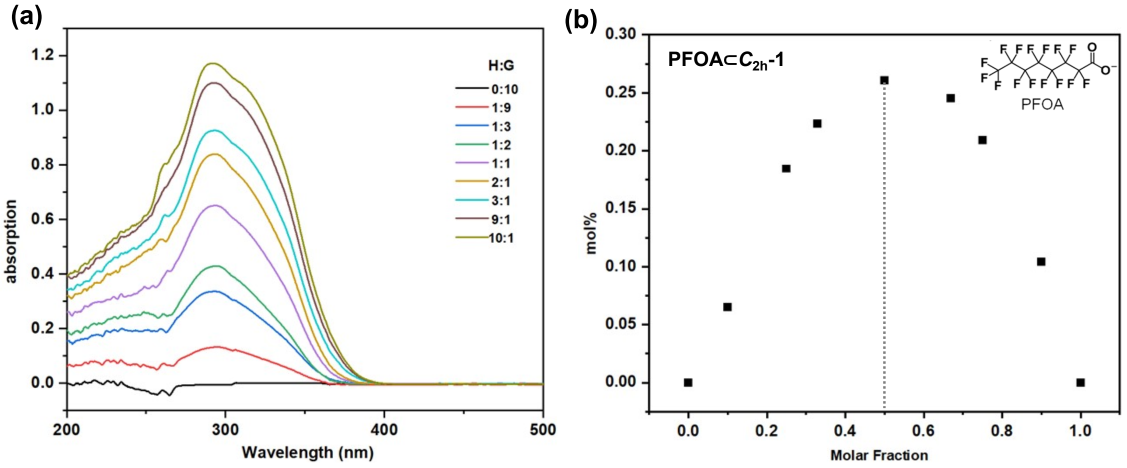


**Figure S33:** (a) UV-vis absorption of metallacage $C_{2h}$-**1** with PFOA in different molar ratios ([$C_{2h}$-**1**]+[PFOA]=6*10$^{-6}$ mol/L); (b) Job's plot of the PFOA⊂$C_{2h}$-**1** in $CH_3CN$, showing a 1:1 stoichiometry.

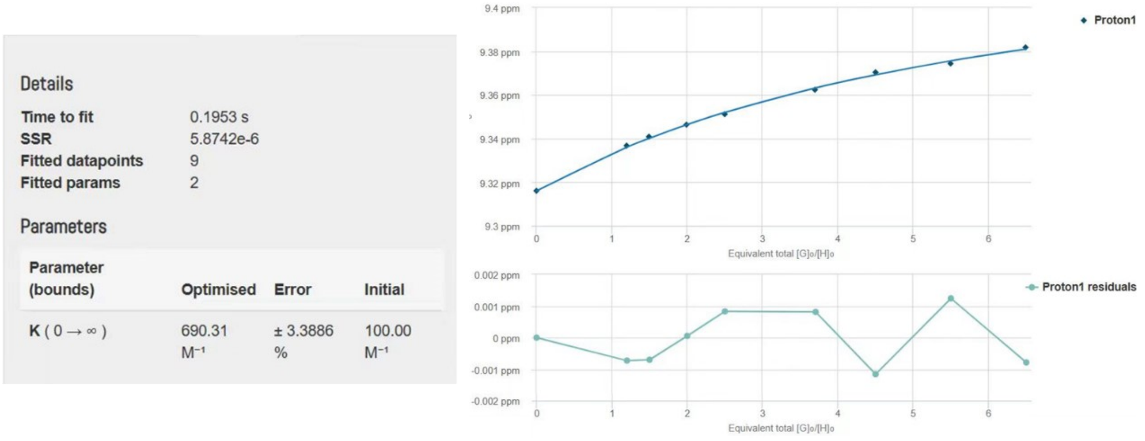


**Figure S34:** Binding isotherms (1:1 system) fitted to the chemical shift of proton signals vs. the equivalents of PFOA added to determine the binding affinity (top); and the residual plot from the fit (bottom).

## 5. Single-crystal X-ray diffraction (XRD)

The single crystals were obtained by slowly diffusing the vapor of isopropyl ether into complexes in acetonitrile for over one month.

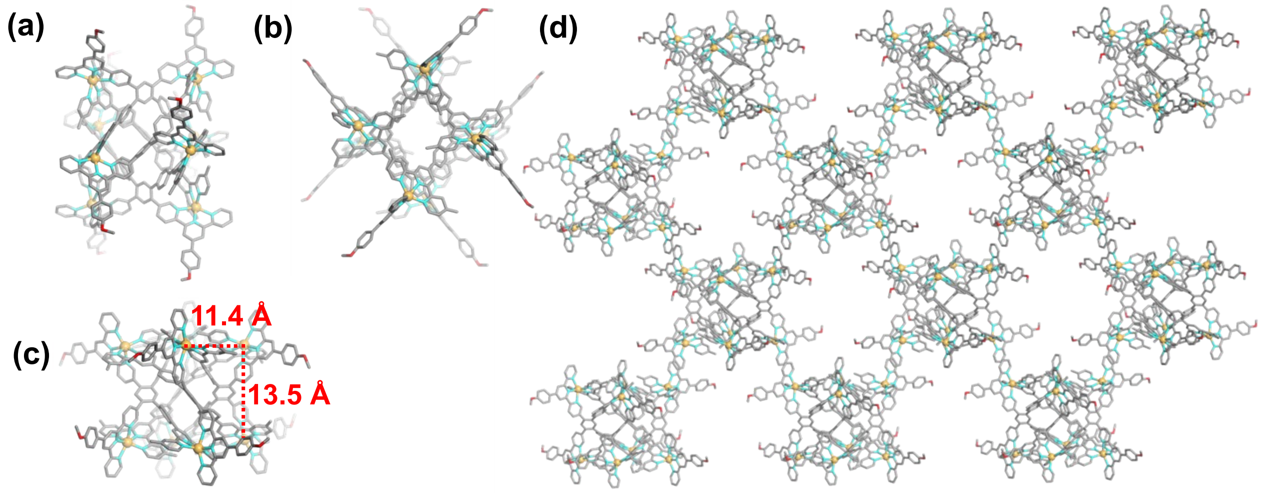


**Figure S35:** (a) The side view, (b) top view and (c) front view of crystal structure of metallo-cage $D_2$-**2**, (d) crystal packing model of metallo-cage $D_2$-**2**.

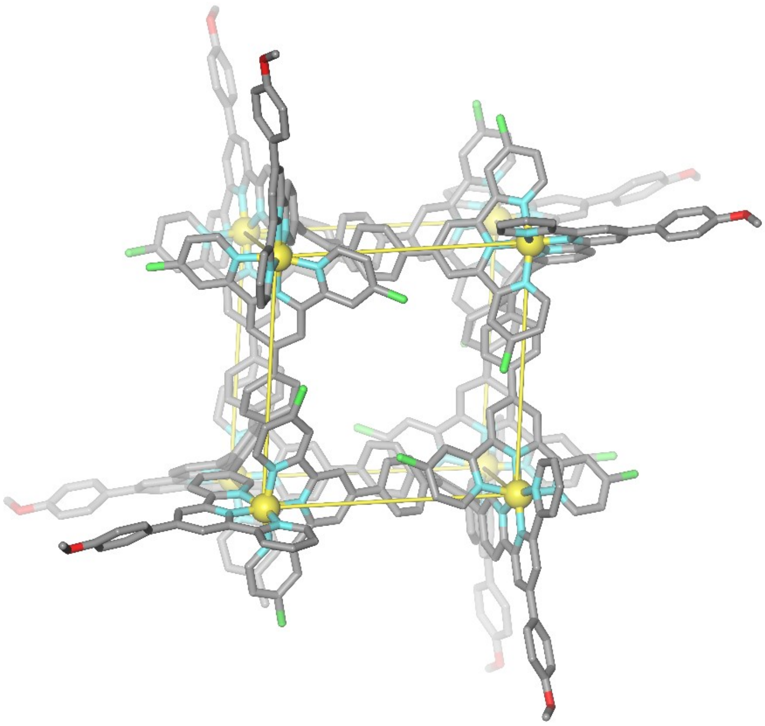

**Figure S36:** The methyl groups of part B (green marked) blocked the pore of $C_{2h}$-**1.**

**Table S1.** Crystal data and structure refinement for metallo-cube $D_2$-**2.**

| | |
|---|---|
| Identification code | $D_2$-**2** |
| Empirical formula | $C_{416}Cd_8F_{96}N_{64}O_{72}S_{32}$ |
| Formula weight | 11076.20 |
| Temperature/K | 101.0 |
| Crystal system | orthorhombic |
| Space group | Ccce |
| a/Å | 28.333(3) |
| b/Å | 43.635(4) |
| c/Å | 40.669(4) |
| α/° | 90 |
| β/° | 90 |
| γ/° | 90 |
| Volume/Å$^3$ | 50280(9) |
| Z | 8 |
| $\rho_{calc}$g/cm$^3$ | 0.872 |
| μ/mm$^{-1}$ | 2.035 |
| F(000) | 13512.0 |
| Radiation | GaKα(λ = 1.34139) |
| 2Θ range for data collection/° | 3.746 to 68.288 |
| Index ranges | -23 ≤ h ≤ 23, -36 ≤ k ≤36, -33 ≤ l ≤ 33 |
| Reflections collected | 127722 |
| Independent reflections | 7630 [$R_{int}$ = 0.0839， $R_{sigma}$ = 0.0416] |
| Data/restraints/parameters | 7630/936/921 |
| Goodness-of-fit on F$^2$ | 1.791 |
| Final R indexes [I>=2σ (I)] | $R_1$ = 0.1328, w$R_2$ = 0.3859 |
| Final R indexes [all data] | $R_1$ = 0.1563, w$R_2$ = 0.4148 |
| Largest diff. peak/hole / e Å$^{-3}$ | 1.61/-0.53 |

## 6. Volume Calculations

In order to determine the available void spaces of $C_{2h}$-**1**, $D_2$-**2**, VOIDOO calculations based on the simulated energy optimal structure (for $C_{2h}$-**1**) and crystal structures (for $D_2$-**2**) were performed.[S3] A virtual probe with a radius of 1.4 Å (set by default, water-sized) was employed, and the standard parameters tabulated below were used.

Maximum number of volume-refinement cycles: 30

Minimum size of secondary grid: 3

Grid for plot files: 0.1

Primary grid spacing: 0.1

Plot grid spacing: 0.1

The cavity volumes were calculated to be 1263 and 867 Å$^3$ for $C_{2h}$-**1**, $D_2$-**2**, respectively.

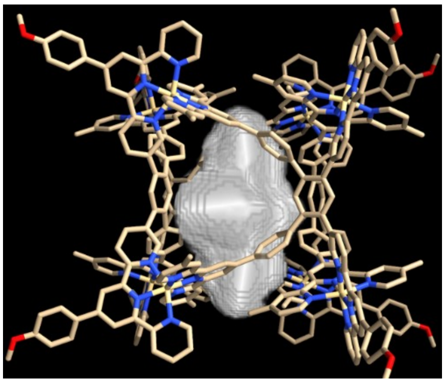

**Figure S37:** VOIDOO calculated void space within the crystal structure of $C_{2h}$-**1**. The volume is calculated to be 1263 Å$^3$.

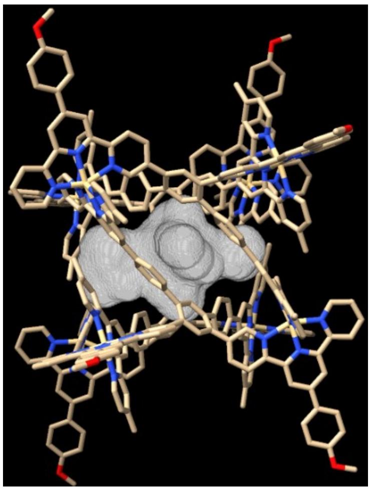

**Figure S38:** VOIDOO calculated void space within the crystal structure of $D_2$-**2**. The volume is calculated to be 867 Å$^3$.

## 7. Calculation

Molecular level understanding on the kind of interaction of the host with one conformer while the other conformer having less interaction with the guest has been checked. As shown in Figure 40, part of the fluorine atoms in guest PFOA interacted with hydrogen atoms in conformer $D_2$-**2** through CH…F hydrogen bonds with about 2.7-3.4 Å distance (about 6 CH…F hydrogen bonds), indicating low bond energy. In terms of PFOA⊂$C_{2h}$-**1** host-guest complex, all of the fluorine atoms in guest PFOA interacted with hydrogen atoms in conformer $C_{2h}$-**1** through CH…F hydrogen bonds with about 2.1-3.1 Å distance (about 11 CH…F hydrogen bonds), showing strong bonding energy (Figure 41). So, the difference of hydrogen bonds between hosts and guest PFOA is the driving force for encapsulation of the guest in conformer $C_{2h}$-**1** rather than conformer $D_2$-**2**.

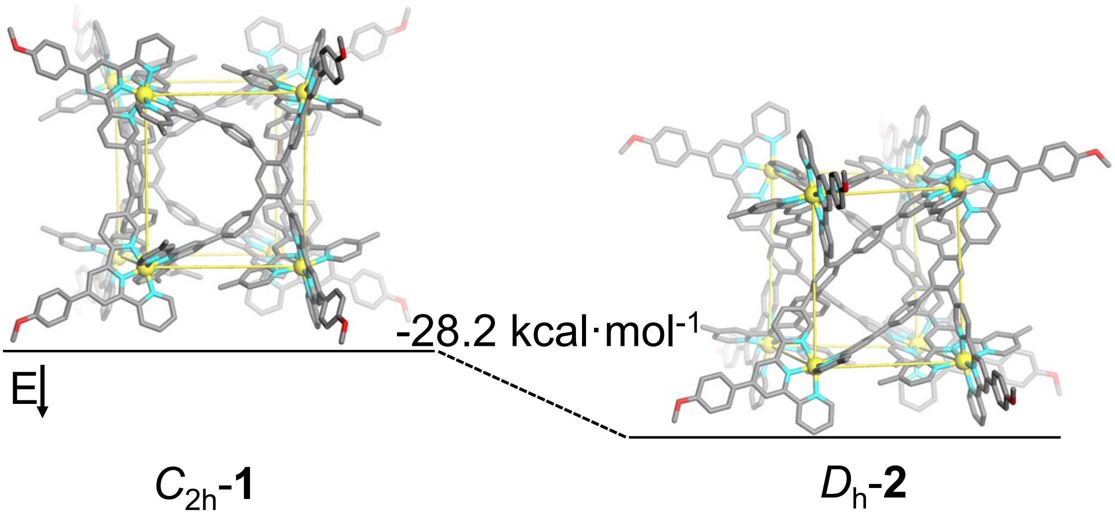


**Figure S39:** The energy-minimized structure of $C_{2h}$-**1**, single-crystal structure of $D_2$-**2**. The energy difference between two structures is presented.

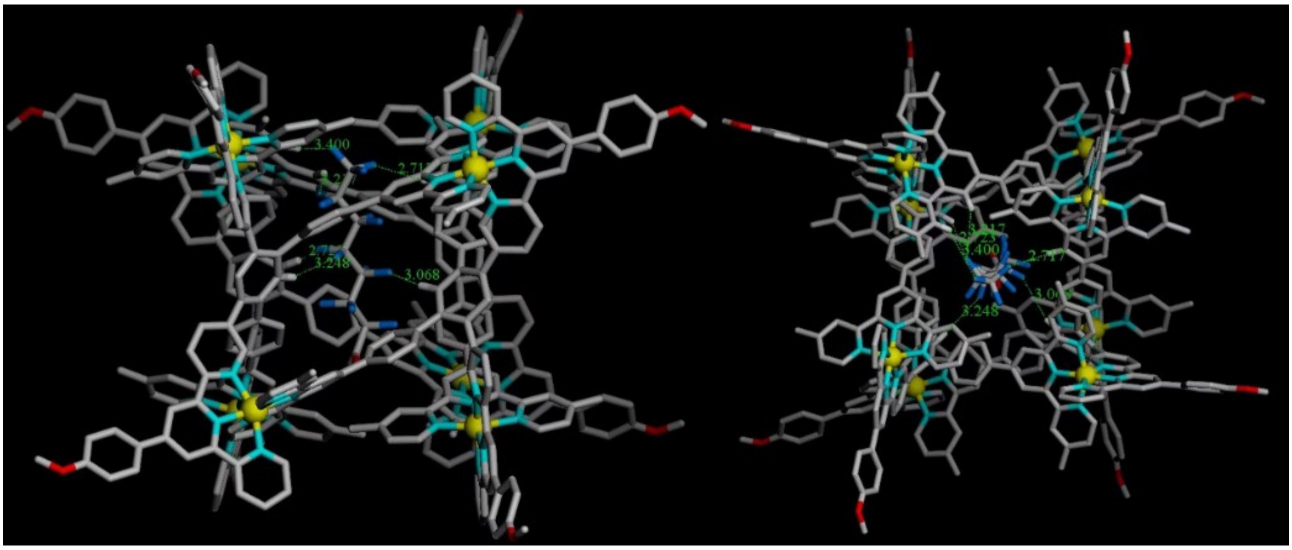

**Figure S40:** CH…F hydrogen bonds in PFOA⊂$D_2$-**2**.

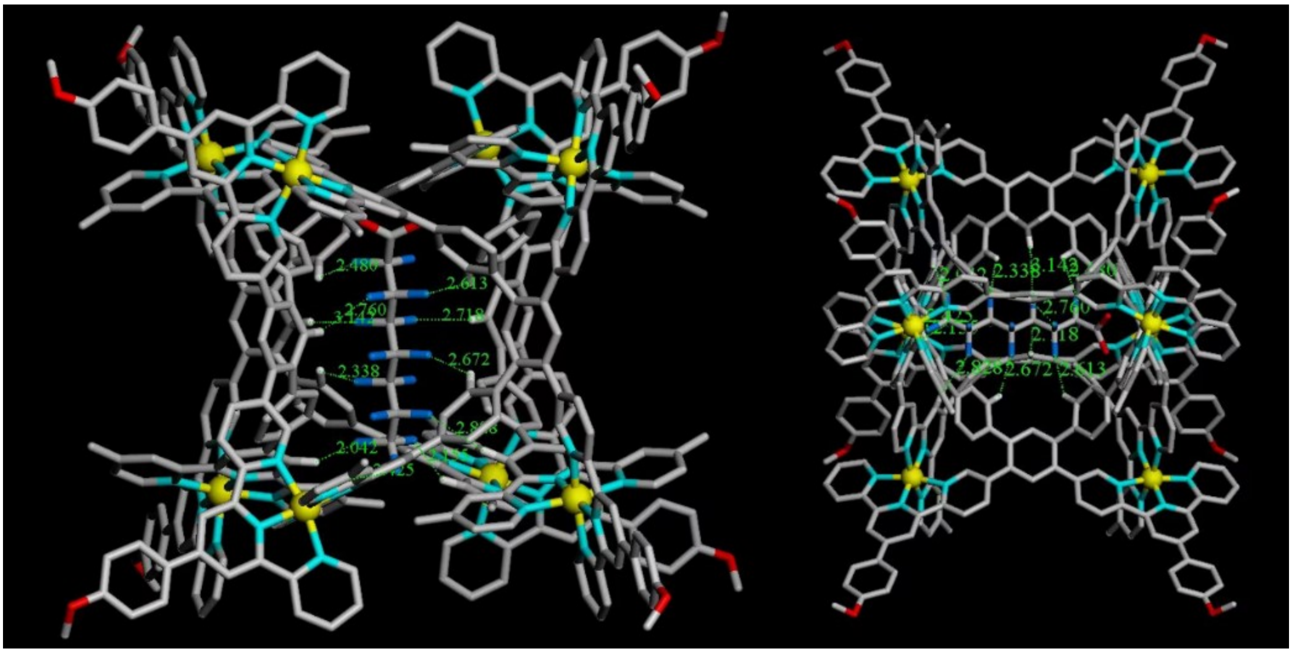

**Figure S41:** CH…F hydrogen bonds in PFOA⊂$C_{2h}$-**1**.

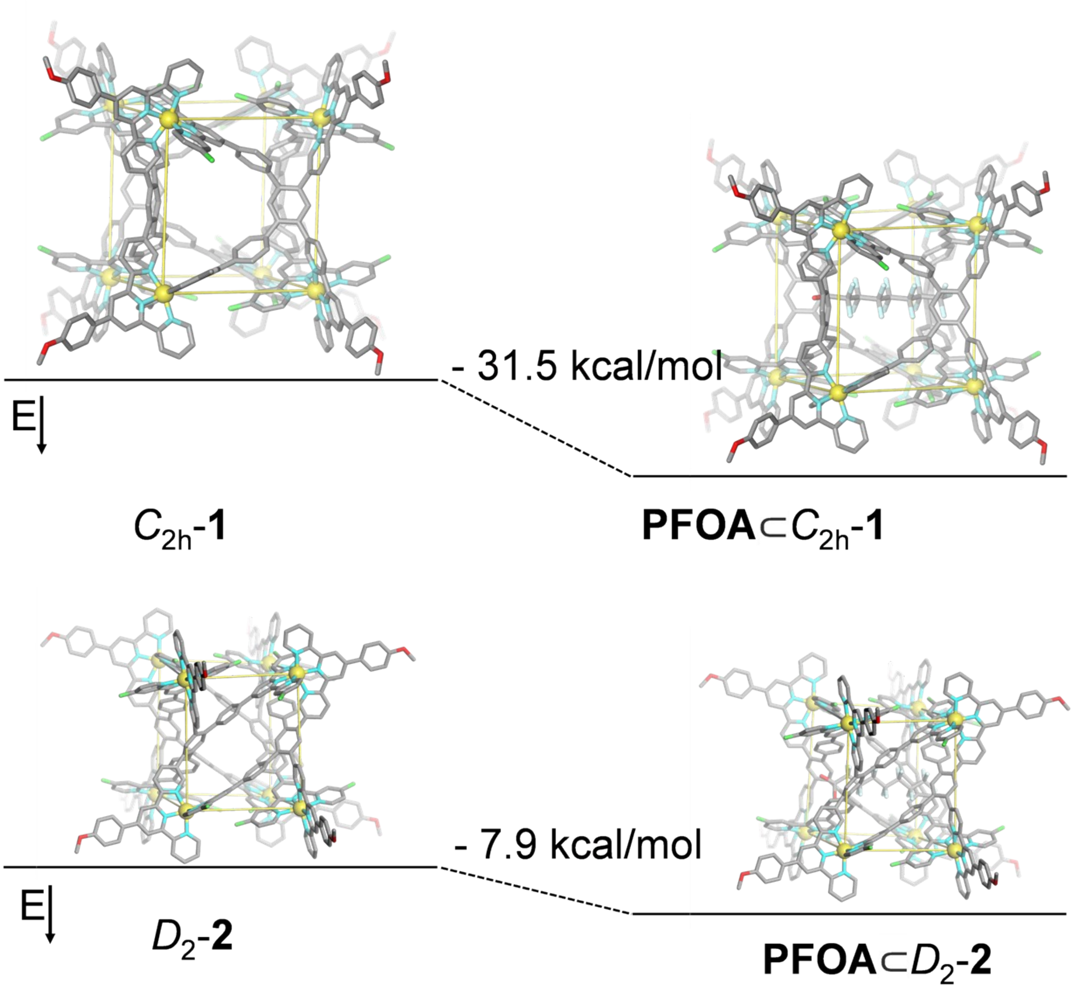


**Figure S42:** The binding energies of host-guest complex PFOA⊂$C_{2h}$-**1** and PFOA⊂$D_2$-**2**.

## 8. The NMR and MS spectra date of $Zn_8L_4$

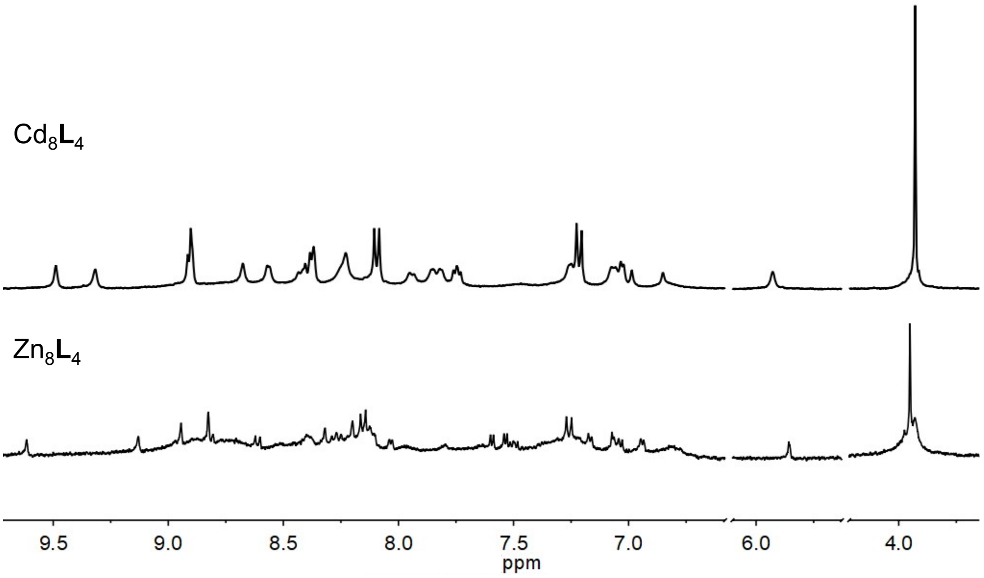


**Figure S43:** The comparison of $^{1}$H NMR spectra of metallo-cage $Cd_8\mathbf{L}_4$ and $Zn_8\mathbf{L}_4$ (500 MHz, 298 K, $CD_3CN$-$d_3$).

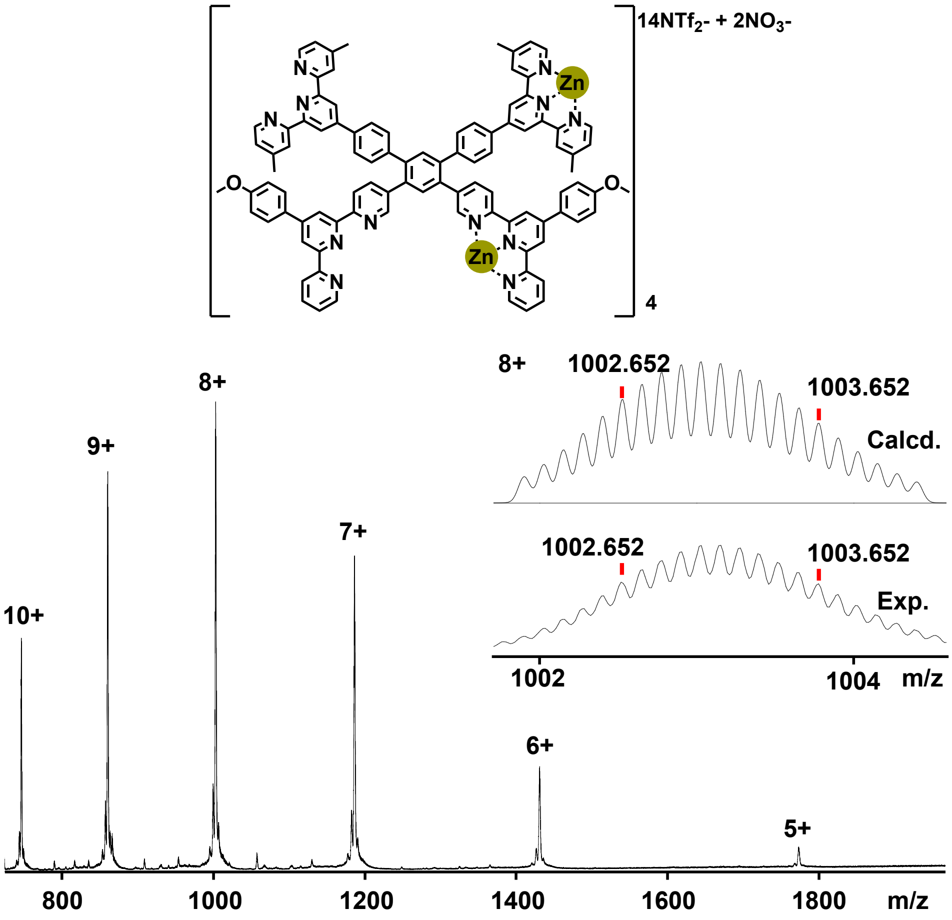


**Figure S44:** ESI-MS spectra of metallo-cage $Zn_8\mathbf{L}_4$ with insert isotopic patter of charge state 8+.

## 9. References


S1. Y. Liu, Z. Jiang, Y. Guan, Q. Bai, Z. Zhang, Y. Li, H. Zhao, T.-Z. Xie, M. Wang, P. Wang and T. Wu, Halide ion directed templation effect of quadruple-stranded helicates, *Cell Rep. Phys. Sci.*, 2022, **3**, 101056.

S2. J. Wang, Z. Jiang, J.-F. Yin, H. Zhao, Q. Dong, K. Li, W. Zhong, D. Liu, J. Yuan, P. Yin, Y. Li, Y. Lin, M. Chen and P. Wang, Strain-Induced Heteromorphosis Multi-Cavity Cages: Tension-Driven Self-Expansion Strategy for Controllable Enhancement of Complexity in Supramolecular Assembly, *Angew. Chem. Int. Ed.*, 2024**, 63**, e202317674.

S3. C. Xu, Q. Lin, C. Shan, X. Han, H. Wang, H. Wang, W. Zhang, Z. Chen, C. Guo, Y. Xie, X. Yu, B. Song, H. Song, L. Wojtas and X. Li, Metallo-Supramolecular Octahedral Cages with Three Types of Chirality towards Spontaneous Resolution, *Angew. Chem. Int. Ed.*, 2022**, 61***,* e202203099.

S4. O. V. Dolomanov, L. J. Bourhis, R. J. Gildea, J. A. K. Howard and H. Puschmann, link to html
5 OLEX2: a complete structure solution, refinement and analysis program, *J. Appl. Cryst.* 2009, **42**, 339–341.

S5. G. M. Sheldrick, SHELXT – Integrated space-group and crystal-structure determination, *Acta Cryst. A*. 2015, **71**, 3–8.

S6. G. M. Sheldrick, Crystal structure refinement with SHELXL, *Acta Cryst. C*. 2015, **71**, 3–8.

S7. P. van der Sluis and A. L. Speck, BYPASS: an effective method for the refinement of crystal structures containing disordered solvent regions, *Acta Cryst. A*. 1990, **46**, 194−201.

S8. A. L. Spek, Single-crystal structure validation with the program PLATON, *J. Appl. Cryst.* 2003, **36**, 7-13.

S9: P. Pachfule, Y. Chen, S. C. Sahoo, J. Jiang, R. Banerjee, Structural Isomerism and Effect of Fluorination on Gas Adsorption in Copper-Tetrazolate Based Metal Organic Frameworks. *Chem. Mater.* 2011, **23**, 2908−2916.

S10. D. E. Coupry, M. A. Addicoat, T. Heine, Extension of the Universal Force Field for Metal–Organic Frameworks. *J. Chem. Theory Comput.* 2016, **12**, 5215−5225.
